# The physiological component of the BOLD signal: Impact of age and heart rate variability biofeedback training

**DOI:** 10.1162/IMAG.a.99

**Published:** 2025-08-07

**Authors:** Richard Song, Jungwon Min, Shiyu Wang, Sarah E. Goodale, Kimberly Rogge-Obando, Ruoqi Yang, Hyun Joo Yoo, Kaoru Nashiro, Jingyuan E. Chen, Mara Mather, Catie Chang

**Affiliations:** Department of Computer Science, Vanderbilt University, Nashville, TN, USA; Program in Neuroscience, Vanderbilt University, Nashville, TN, USA; Leonard Davis School of Gerontology, University of Southern California, Los Angeles, CA, United States; Department of Biomedical Engineering, Vanderbilt University, Nashville, TN, USA; Athinoula A. Martinos Center for Biomedical Imaging, Massachusetts General Hospital, Boston, MA, United States; Department of Radiology, Harvard Medical School, Boston, MA, United States; Department of Psychology, University of Southern California, Los Angeles, CA, United States; Department of Biomedical Engineering, University of Southern California, Los Angeles, CA, United States; Department of Electrical and Computer Engineering, Vanderbilt University, Nashville, TN, United States

**Keywords:** aging, BOLD fMRI, peripheral physiological signals, heart rate variability, autonomic nervous system, central autonomic network

## Abstract

Aging is associated with declines in autonomic nervous system (ANS) function, impaired neurovascular coupling, and diminished cerebrovascular responsiveness—factors that may contribute to cognitive decline and neurodegenerative diseases. Understanding how aging alters the integration of physiological signals in the brain is crucial for identifying potential interventions to promote brain health. This study examines age-related differences in coupling between low-frequency cardiac rate and respiratory volume fluctuations and the blood oxygenation level-dependent (BOLD) signal, using two independent resting-state fMRI datasets with concurrent physiological recordings from younger and older adults. Our findings reveal significant age-related reductions in the percent variance of the BOLD signal explained by heart rate (HR), respiratory variation (RV), and end-tidal CO^2^, particularly in regions involved in autonomic regulation, including the orbitofrontal cortex, anterior cingulate cortex, insula, basal ganglia, and white matter. Cross-correlation analysis also revealed that younger adults exhibited stronger HR–BOLD coupling in white matter, as well as a more rapid BOLD response to RV and CO^2^ in gray matter. Additionally, we investigated the effects of heart rate variability biofeedback (HRV-BF) training, a non-invasive intervention designed to modulate heart rate oscillations. The intervention modulated physiological–BOLD coupling in a manner dependent on both age and training condition: older adults who underwent HRV-BF to enhance HR oscillations exhibited a shift toward younger-like HR–BOLD coupling patterns. These findings suggest that HRV-BF may help mitigate age-related declines in autonomic or cerebrovascular function. Overall, this study underscores the role of physiological dynamics in brain aging and highlights the importance of considering autonomic function when interpreting BOLD signals. By demonstrating that HRV-BF can modulate physiological–BOLD interactions, our findings suggest a potential pathway for enhancing cerebrovascular function and preserving brain health across the lifespan.

## 1 Introduction

The autonomic nervous system (ANS) is a sub-branch of the peripheral nervous system that is responsible for regulating physiological functions of the body, including heart rate and respiration. It is well documented, however, that ANS health declines with age (Mather, 2024; Olivieri et al., 2024; Takla et al., 2023; Thayer et al., 2010). For example, aging is associated with decreased heart rate variability (HRV), which reflects the natural ability of the brain to modulate oscillations in heart rate (Britton et al., 2007; Jandackova et al., 2016; Mather, 2024; Reardon & Malik, 1996; Thayer et al., 2010; Zulfiqar et al., 2010). As the ANS controls cardiovascular responses, it directly influences cerebral blood flow and the dynamics of neurovascular coupling, which are vital for maintaining a healthy level of blood flow to the brain to sustain cognitive functions (Goadsby, 2013; Koep et al., 2022; Mankoo et al., 2023). With aging, the decline in ANS health can compromise the ability of the brain to regulate cerebral blood flow efficiently (Lu et al., 2011). This diminished neural–vascular regulation, exacerbated by age-related structural changes in the vasculature, has been linked to cognitive decline and diseases such as Alzheimer’s and stroke (Han et al., 2021; Mankoo et al., 2023; Sweeney et al., 2019).

Functional magnetic resonance imaging (fMRI) is a non-invasive technique for measuring brain activity by detecting changes in local blood oxygenation levels. Since fMRI relies on the hemodynamic response, the blood oxygenation level-dependent (BOLD) signal is affected by low-frequency fluctuations in peripheral physiological processes, including natural variations in respiratory variation (RV) and heart rate (HR) (Murphy et al., 2013; Wise et al., 2004). In fMRI studies, it is common to regress out RV and HR from the BOLD signal, as these effects introduce non-neuronal fluctuations that may confound inferences about neural activity. However, several new avenues of research have indicated that the physiological component of the fMRI BOLD signal may provide valuable information related to ANS and cerebrovascular health (Bright et al., 2020; Donahue et al., 2016; Makedonov et al., 2013; Mather & Thayer, 2018; Yang et al., 2022). For instance, areas of the brain associated with regulating naturalistic breathing rhythms were found to exhibit strong BOLD–RV coupling (Chen et al., 2020), and regions demonstrating high BOLD–HR coupling were found in regions of high vessel density (Chen et al., 2020).

Due to the deterioration of ANS health with age, the effect of age on physiological–BOLD coupling is of interest. The joint dynamics of physiological signals (e.g., HR and RV) and the BOLD signal may shed light on age-related changes in autonomic and cerebrovascular health, potentially uncovering early indicators of neurodegenerative or cardiovascular conditions. One study demonstrated that aging was associated with lower levels of resting-state BOLD variability and that cardiovascular health moderated this effect (Tsvetanov et al., 2021). Another study demonstrated that the hemodynamic response was smaller and slower in older adults than in younger adults when performing audio/visual sensorimotor tasks (West et al., 2019). Further, animal models have revealed that aging may reduce neurovascular coupling due to structural changes in neurovasculature, such as vascular rarefaction and endothelial dysfunction, suggesting that the relationship between BOLD and physiological signals may be an indicator of aging (Yabluchanskiy et al., 2021). However, it is presently unclear how the spatiotemporal association of BOLD fMRI signals with low-frequency breathing and heart rate variability changes with aging.

Here, we identify age-related differences in the propagation of spontaneous heart rate and respiratory fluctuations into resting-state BOLD signals. Differences between older and younger adults prominently included brain regions that have been implicated in autonomic regulation. Additionally, we demonstrate that HRV biofeedback training, a non-invasive paced breathing technique to modulate HR oscillations, alters the dynamics of BOLD–physiological coupling in older adults to more closely resemble patterns observed in younger adults. Overall, this research aims to advance our understanding of how physiological signals manifest within the spatial and temporal patterns of the BOLD response, while also investigating how both the aging process and HRV biofeedback training may modulate these underlying dynamics.

## 2 Methods

### 2.1 Datasets

We included resting-state fMRI scans with high-quality physiological data from 399 participants in the Nathan Kline Institute (NKI) Rockland sample (Nooner et al., 2012). Data were examined from a younger (range: 19–36, mean: 25.98, std: 4.72, n = 144) and an older (range: 50–85, mean: 63.00, std: 8.28, n = 255) group. The NKI institutional review board approved data collection, and participants gave informed consent. An MPRAGE sequence was used to retrieve an anatomical image for each subject (TR/TE = 1900/2.52 ms; flip angle = 9°; slice thickness = 1.0 mm; number of slices = 192; matrix = 256 × 256; field of view = 250 mm). Resting-state scans were acquired using an EPI sequence (TR = 1400 ms; duration 9.4 min; 404 volumes; voxel size = 2.0 x 2.0 x 2.0 mm; flip angle = 65°; field of view= 224 mm). During the scan, physiological data sampled at 62.5 Hz were also collected from participants. Cardiac data were collected via photoplethysmogram (PPG) and respiration was measured using a respiration belt.

We also included resting-state fMRI scans with high-quality physiological data from 110 participants in the Heart Rate Variability and Emotional Regulation (HRV-ER) dataset (Yoo et al., 2023), who were either in a younger (range: 18–30, mean: 22.19, std: 2.86, n = 59) or an older (range: 55–80, mean: 64.69, std: 6.36, n = 51) group. Resting-state fMRI was acquired using a multi-echo-planar imaging sequence (TR = 2.4 seconds; TE 18/35/53 ms; slice thickness = 3.0 mm; flip angle = 75°; field of view = 240 mm; voxel size = 3.0 × 3.0 × 3.0 mm; 175 volumes; duration 7 min). In-scan cardiac data were collected using PPG, CO_2_ levels were collected using capnography, and respiration was measured using a breathing belt. Physiological data were initially collected at 10 kHz but was downsampled to 1 kHz. Scans were collected from all participants both before and after a 5-week HRV biofeedback training intervention.

During the 5-week HRV biofeedback training, half of the participants completed daily sessions in which they did slow paced breathing while receiving biofeedback to increase their heart rate oscillations (Osc+), while the other half received biofeedback to decrease heart rate oscillations (Osc−). In weekly laboratory sessions, participants in the Osc+ condition practiced breathing at different paces to identify their resonance frequency, aiming to maximize heart rate oscillations using biofeedback from the emWave Pro software. In the Osc− condition, participants used their own strategies to reduce heart rate oscillations, receiving feedback via a “calmness score,” which increased as their heart rate oscillations decreased. In between the weekly laboratory sessions, training was performed twice daily at home for 20 minutes each using the breathing pace (Osc+) or strategy (Osc−) identified as most effective for increasing (Osc+) or reducing (Osc−) heart rate oscillations. Of the 110 participants for which baseline resting-state fMRI scans and in-scanner physiological recordings were obtained, 78 participants (20 younger Osc+, 21 younger Osc−, 19 older Osc+, 18 older Osc−) also had the fMRI scans and physiological recordings data after the 5-week HRV biofeedback training.

Although the NKI dataset contains data from adults between the ages of 37 to 49 years, our decision to omit the 37- to 49-year age range was driven by study design rather than power considerations. The HRV-ER intervention dataset, to which we directly compare the NKI results, contains only younger (18–30 years) and older (55–80 years) participants; aligning the NKI bins with those groups allows a like-for-like validation across independent cohorts and focuses on the life-span segments where differences in autonomic and vascular characteristics are most pronounced.

### 2.2 Imaging data preprocessing

For anatomical data in both NKI and HRV-ER datasets, T1-weighted images were skull-stripped using FSL’s Brain Extraction Tool (BET), followed by intensity non-uniformity correction with AFNI’s *3dUnifize*.

In the NKI dataset, motion correction of the fMRI data was carried out using FSL *mcflirt*. ICA FIX (Griffanti et al., 2014) was additionally used to further correct for motion artifacts including high-frequency physiologically coupled motion artifacts and other MRI acquisition-related artifacts. ICA FIX functions as an independent component classifier, and was trained using data from 25 subjects with a balanced age distribution from the NKI dataset. For training, each independent component was manually labeled as either “noise” or “not noise,” enabling ICA FIX to identify and remove noise components in the remainder of the dataset.

In the HRV-ER dataset, fMRI data underwent motion correction using AFNI’s *3dvolreg*. Motion correction was applied to the second echo, with the resulting motion parameters used to align the first and third echoes via *3dAllineate*. Slice timing correction was performed with AFNI’s *3dTshift*. Multi-echo fMRI data were denoised using *tedana*, which applied multi-echo independent component analysis (ME-ICA) to distinguish BOLD components from non-BOLD artifacts (Kundu et al., 2012, 2017).

In both datasets, spatial normalization of functional data to MNI space was achieved by first aligning functional volumes to the T1-weighted anatomical images with FSL’s *epi_reg*, followed by ANTs-based registration to the MNI152 2 mm template. The ANTs routine *antsApplyTransforms* was used to apply this nonlinear transformation to the T1-registered functional data. Smoothed functional images (3 mm FWHM) were generated using AFNI’s *3dmerge*. Nuisance regression, including fourth-order polynomial detrending, was performed with *3dDetrend*, followed by the addition of the mean signal using *3dTstat*.

### 2.3 Physiological data preprocessing

Physiological data, including heart rate (HR), respiratory variation (RV), and end-tidal CO_2_, were processed to create fMRI regressors aligned with the fMRI time series. To calculate HR, the raw photoplethysmography (PPG) signal was bandpass filtered between 0.5 and 2 Hz using a second-order Butterworth filter to enhance heartbeat clarity for peak detection. Peaks were identified using a minimum peak height threshold of 5% of the interquartile range, and inter-beat intervals (IBI) were calculated. After visual inspection of the IBI time series for artifacts (e.g., due to poor peak detection), we interpolated over any instances where artifacts occurred. Heart rate was calculated as the inverse of the median inter-beat interval (IBI), using 6-second sliding windows centered at each fMRI TR. Metrics of heart rate variability (HRV), such as RMSSD, high-frequency (HF), and low-frequency (LF) power, were also derived from the IBI. The HF power band was defined between 0.15 and 0.4 Hz, and the LF power band was defined between 0.04 and 0.15 Hz (Burr, 2007).

RV was calculated as the temporal standard deviation of the raw respiration waveform within 6-second windows centered at each TR. To account for individual differences in torso size, the RV signal was normalized between -1 and 1 for each participant. RV normalization was performed by first determining lower and upper bounds at the 1.45th and 98.55th percentiles using a histogram-based approach. The mean RV within these bounds was then subtracted to center the data approximately around zero, and this mean was also subtracted from the lower and upper bounds. Finally, positive and negative values were divided by their respective adjusted upper or lower bounds, resulting in a normalized range approximately between -1 and 1.

The HRV-ER dataset also included capnography recordings for measuring CO_2_. To account for time delays in the capnography signal, it was time shifted to optimize alignment with the respiration waveform, reflecting the expected inverse correlation between the two signals. End-tidal CO_2_ values were then extracted from the re-aligned capnography peaks and resampled to match the fMRI TR. For both datasets, breathing rate was also calculated in breaths per minute using the *NeuroKit2* package in Python (Makowski et al., 2021).

### 2.4 Modeling relationships between BOLD and physiological signals

**Fig. 1.**
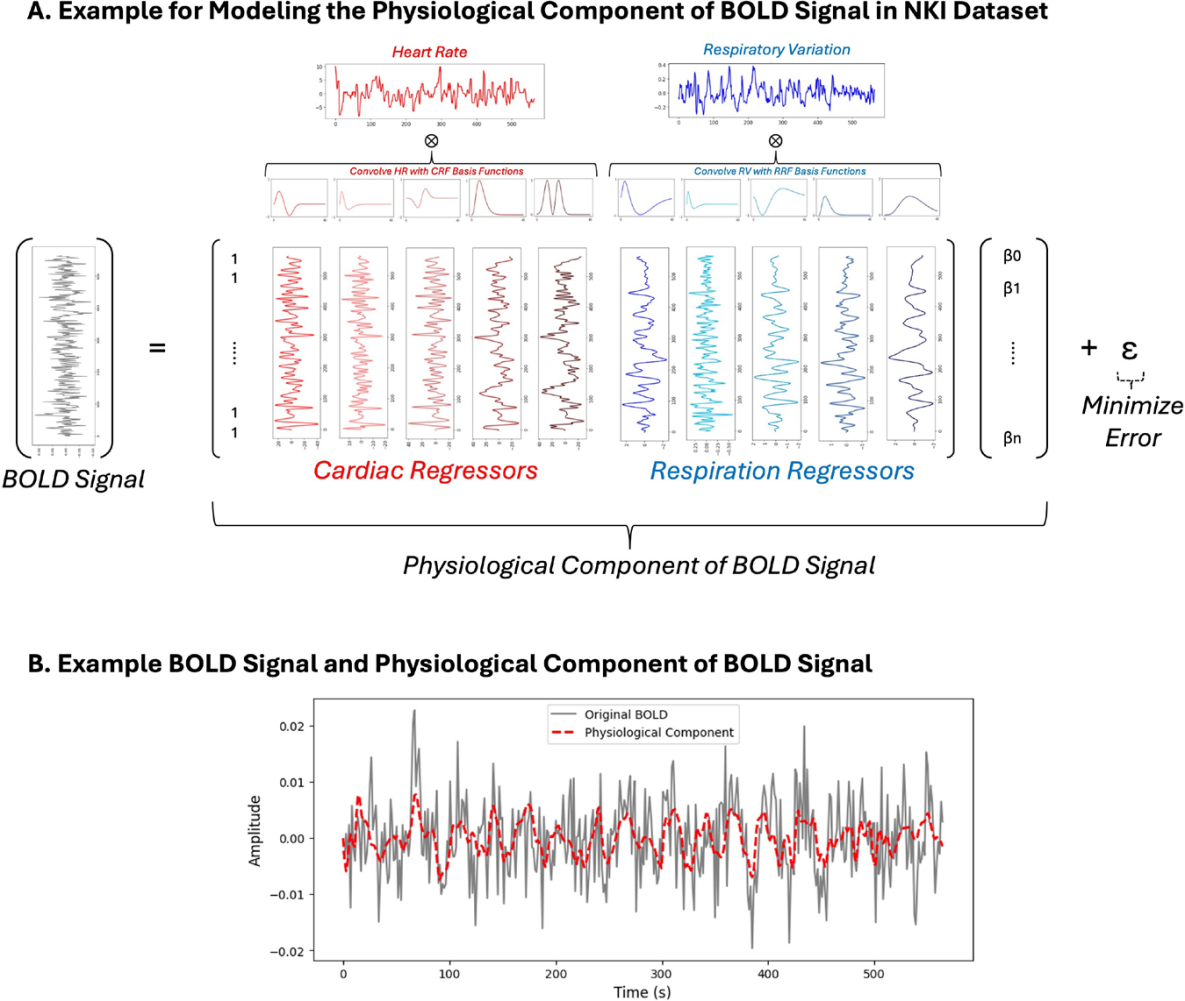
Schematic for the model to determine the physiological component of the BOLD signal. (A) After detrending and normalizing heart rate and respiratory variation, the signals are convolved with CRF and RRF basis functions. For every voxel, a general linear model is used to find beta weights for each of the cardiac and respiration regressors to minimize the error from the original BOLD signal. (B) Example of an original BOLD signal (normalized to percent signal change) and the corresponding physiological component.

The physiological data were used to create regressors for modeling the impact of resting-state physiological fluctuations on BOLD signal variance. To account for respiratory effects, we convolved the RV signal with the primary Respiratory Response Function (RRF) (Birn et al., 2008), as well as temporal and dispersive derivative basis functions of the RRF. This allowed us to model regional differences in the propagation of RV effects on the BOLD signal (Chen et al., 2020). Similarly, heart rate (HR) was convolved with the primary Cardiac Response Function (CRF) (Chang et al., 2009) and its temporal and spatial derivative basis functions. The RRF and CRF model the physiological impulse responses, capturing how RV and HR fluctuations influence the BOLD signal. Figure 1 presents a schematic overview of the model.

In the HRV-ER dataset, we also modeled the impact of end-tidal CO_2_ on the BOLD signal by convolving it with a previously parameterized double-gamma end-tidal CO_2_ response function (Golestani et al., 2015), and its temporal derivative. The Golestani et al. (2015) basis functions were chosen to model end-tidal CO_2_ response over other commonly used response functions for modeling the resting-state BOLD response to CO_2_, such as the canonical hemodynamic response function (Yao et al., 2021), due to its ability to better capture the delayed response of the BOLD signal in older adults to CO_2_ compared with younger adults, which is detailed more in Section 3.3 and Supplementary Figure 1.

For each dataset, we fitted three separate linear models to assess the effect of physiological activity on the BOLD signal: (1) a model with both cardiac and respiration regressors, (2) a model with only cardiac regressors, and (3) a model with only respiration regressors. These models provided the BOLD signal variance explained by both physiological factors combined, cardiac activity alone, and respiration alone, respectively, for each voxel across all participants. Average response function profiles for both older and younger adults using the joint cardiac and respiration model are shown in Supplementary Figure 2. In practice, shared variance between cardiac and respiratory fluctuations may be captured non-specifically in both models (Shmueli et al., 2007). Therefore, the explained variance in models (2) and (3) may include overlapping components that are not orthogonal to each other or to the joint model (1).

**Fig. 2.**
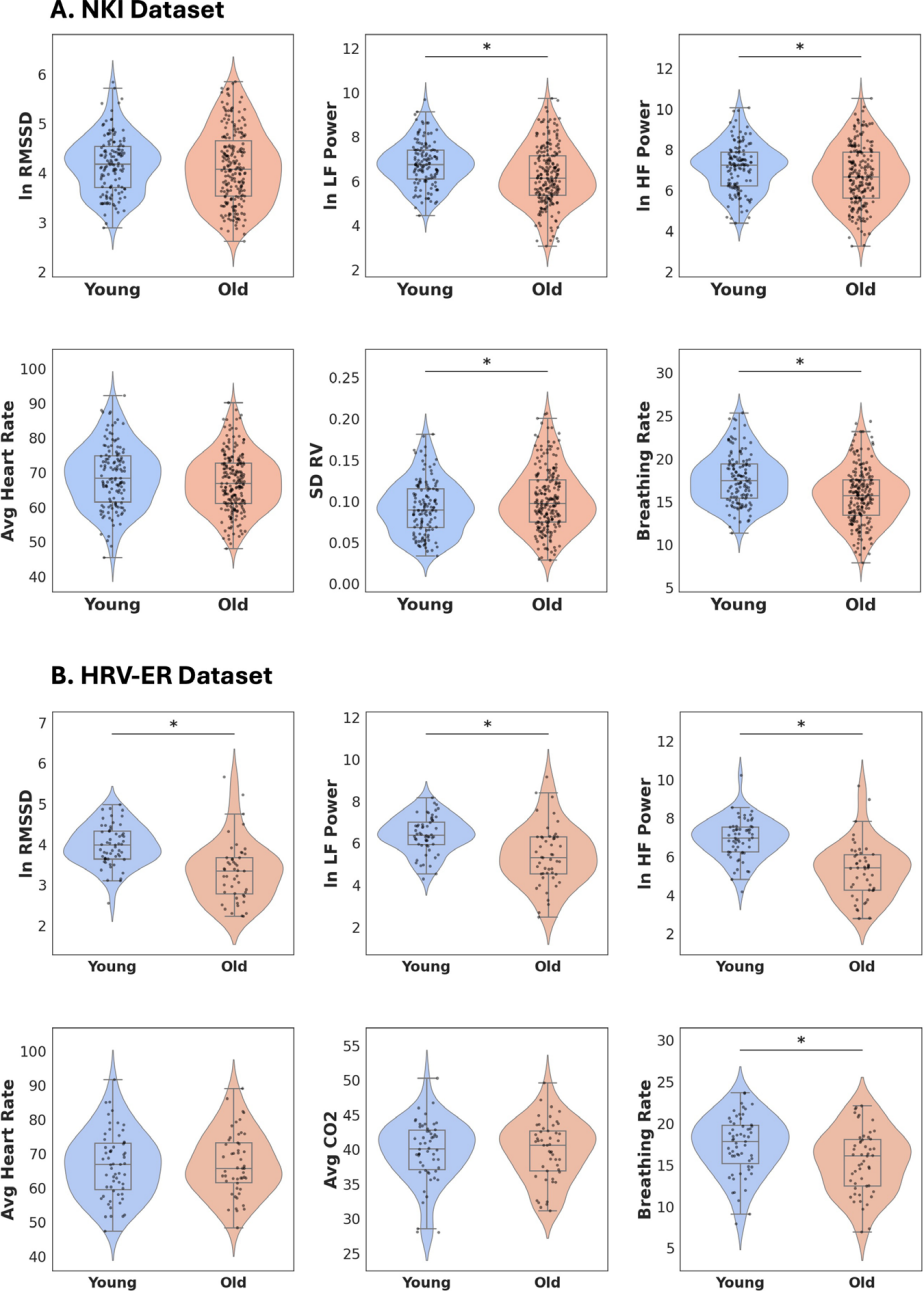
Log-transformed metrics of HRV (RMSSD, LF Power, HF Power) and low-frequency peripheral physiology across the (A) NKI dataset and (B) HRV-ER dataset. Standard box and whisker plots are overlaid over the violin plots. *p < 0.05.

Since the signal quality for respiration belt data collected in the HRV-ER dataset was deemed poor after visual inspection, we used end-tidal CO_2_ as the measure of respiration in this dataset. In the NKI dataset, RV was used as the measure of respiration. Both datasets used heart rate as the primary cardiac measure. To determine the relative contribution of cardiac and respiratory activity to the BOLD signal, we calculated the percent variance explained (PVE) by dividing the variance in the BOLD signal explained by each model by the total variance in the original BOLD signal for every voxel.

In addition to the PVE analysis, whole-brain voxel-wise cross-correlations were computed to examine the temporal relationship between physiological signals and BOLD fluctuations. Cross-correlations were calculated between the BOLD signal and HR/RV for the NKI dataset and between the BOLD signal and HR/end-tidal CO_2_ for the HRV-ER dataset. The RV, HR, and end-tidal CO_2_ signals were not convolved with response functions prior to the cross-correlation analysis; therefore, the cross-correlation analysis makes no assumptions about the form of the dynamic association between physiological and fMRI signals. These correlations were computed across time lags ranging from -2.8 seconds to 21.4 seconds in the NKI dataset, with 1.4 seconds increments, and from -2.4 seconds to 21.6 seconds in the HRV-ER dataset, with 2.4 seconds increments, corresponding to the respective TRs of each dataset.

To further capture global trends at a macro level, cross-correlations were also computed using the BOLD signal averaged across three major tissue compartments: gray matter, white matter, and ventricles. This approach allowed for the examination of broader patterns in physiological signal propagation beyond voxel-level resolution. Statistical comparisons were conducted at each time lag using t-tests, with multiple comparisons corrected using Bonferroni correction.

### 2.5 Whole-brain statistical testing

Whole-brain maps of physiological signal propagation into BOLD fMRI data were compared in older versus younger adults using the HRV-ER and NKI datasets, focusing on the baseline (pre-intervention) condition in the HRV-ER dataset. Statistical differences in percent variance explained maps, or voxel-wise cross-correlation maps, were assessed using two-sample t-tests. To evaluate the effects of the HRV biofeedback intervention, pre- and post-intervention data from the subset of HRV-ER participants with both conditions were used to compute post- minus pre-differences separately for younger and older adults within each intervention condition (Osc+ and Osc−). To assess the effect of age on the intervention response, these post–pre difference maps were then compared between age groups using two-sample t-tests. For all statistical analyses of whole-brain maps, multiple comparisons were corrected using the Threshold-Free Cluster Enhancement (TFCE) algorithm with 5000 permutations. This approach computes the null distribution empirically by shuffling group labels while preserving group sizes, ensuring that no parametric assumptions about equal variances or normality are required. As such, the validity and unbiasedness of the test are preserved even when group sizes differ, because permutation tests inherently maintain the original sampling structure.

## 3 Results

### 3.1 Physiological differences by age

Information about physiological metrics in the participants of the two datasets can be found in Table 1 and Table 2, and is summarized in Figure 2. When calculating summary statistics, we identified outliers using the interquartile range (IQR) method, where values falling below Q1-1.5 × IQR or above Q3 + 1.5 × IQR were excluded from analysis for that specific metric only, while retaining the participant’s valid data for other measurements. Since RMSSD, low frequency (LF) HRV, and high frequency (HF) HRV are not normally distributed, we applied a natural log transformation before statistical analysis. As detailed in Table 1 and Table 2, the older adults exhibited significantly lower ln(LF HRV) and ln(HF HRV) than younger adults. Although older adults had lower RMSSD than younger adults, this difference was not significant in the NKI dataset, although it was in the HRV-ER dataset. As shown in Table 1, older adults also exhibited higher standard deviation in RV than younger adults. However, in both datasets, average HR was not significantly different between the two age groups. In addition, breathing rate was significantly higher in younger adults in both datasets.

**Table 1.** Physiological differences by age in the NKI dataset.

Metric	Younger adults (n = 144)	Older adults (n = 255)	t-Statistic	*p*-value
ln RMSSD	4.197 (0.410)	4.186 (0.640)	0.144	0.885
ln LF	6.879 (1.239)	6.373 (2.211)	3.553**	4.266e-04
ln HF	7.177 (1.661)	6.814 (2.608)	2.310*	2.142e-02
Avg HR	69.046 (82.872)	67.442 (80.538)	1.696	9.062e-02
SD RV	0.094 (0.001)	0.104 (0.002)	-2.551*	1.115e-02
Breathing rate	17.700 (9.253)	15.610 (11.803)	5.922**	7.212e-09

Mean and variance for all measures are shown, along with t-test statistics comparing averages across age groups.

*p < 0.05, **p < 0.001.

**Table 2.** Physiological differences in the HRV-ER dataset by age and HRV biofeedback condition (Osc+ vs. Osc−).

	Younger Osc+ (n = 20)			Young Osc− (n = 21)			Old Osc+ (n = 19)			Old Osc− (n = 18)			Young vs. old pre t-stats
Metric	Pre	Post	t-stat	Pre	Post	t-stat	Pre	Post	t-stat	Pre	Post	t-stat	t-stat
ln RMSSD	3.89 (0.36)	3.98 (0.36)	-0.985	4.06 (0.31)	4.05 (0.22)	0.049	3.13 (0.39)	3.53 (0.52)	-1.397	3.42 (0.72)	3.14 (0.68)	1.105	5.140***
ln LF	6.53 (0.78)	6.73 (0.72)	-0.938	6.30 (0.75)	6.49 (1.11)	-0.902	5.30 (1.65)	6.01 (2.30)	-1.699	5.82 (1.79)	5.56 (2.64)	0.609	4.301***
ln HF	6.71 (1.20)	7.00 (1.43)	-1.283	7.04 (1.26)	7.02 (0.87)	0.072	4.82 (1.34)	5.87 (1.64)	-2.371*	5.78 (2.44)	5.49 (2.48)	0.553	6.495***
Avg HR	67.64 (102.85)	66.65 (79.07)	0.561	66.93 (82.67)	65.91 (63.38)	0.589	65.68 (39.37)	62.90 (65.45)	2.233*	69.00 (105.87)	68.04 (100.84)	0.721	-0.153
Avg CO_2_	38.48 (25.69)	39.30 (13.53)	-0.574	39.53 (22.44)	39.10 (35.22)	0.290	39.74 (14.10)	39.30 (18.29)	0.607	40.68 (12.82)	42.08 (16.28)	-2.753*	-0.019
Breathing Rate	16.90 (16.08)	15.50 (19.07)	1.744	18.03 (7.60)	18.13 (7.53)	-0.212	15.87 (11.98)	14.86 (17.80)	1.586	13.73 (13.59)	13.66 (10.36)	0.104	2.767**

Mean and variance for all measures are shown, along with t-test statistics comparing averages across age groups and between pre- and post-intervention within each group.

*p < 0.05, **p < 0.001, ***p < 0.001.

### 3.2 Age differences in the percent variance of the BOLD signal explained by peripheral physiological measures

To examine the extent to which the fMRI BOLD signal was associated with peripheral physiological measures, we convolved heart rate and respiration time courses with their respective physiological impulse response basis functions to obtain cardiac and respiration regressors, which we then fit to the BOLD signal in each voxel using a general linear model (Section 2.4). For the comparison of age-related differences in the HRV-ER dataset, subjects’ percent variance explained maps were calculated based only on their baseline scan prior to the HRV biofeedback intervention.

**Fig. 3.**
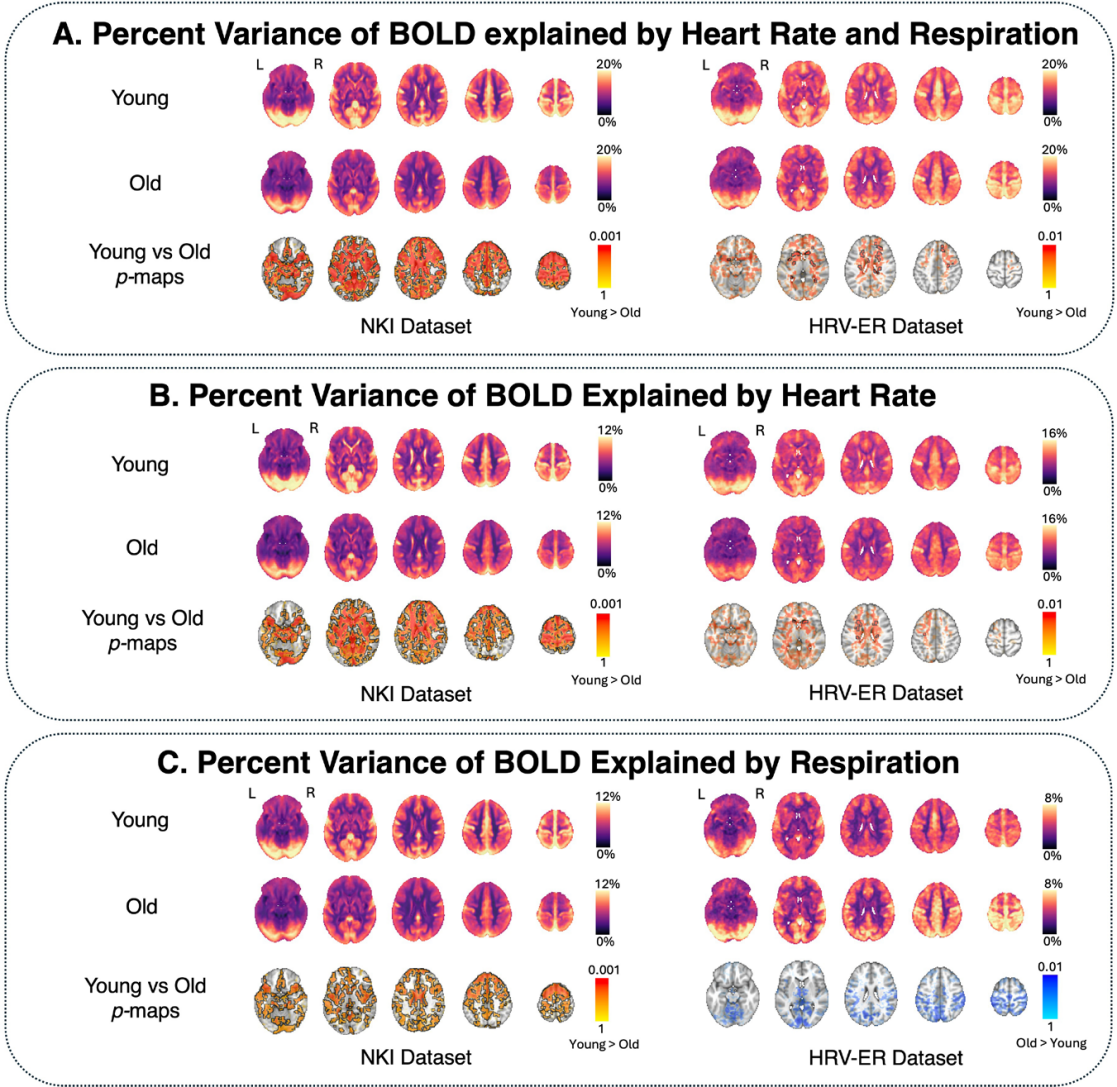
Percent variance of BOLD signal explained by low-frequency peripheral physiology across all voxels. Three separate models were used to determine the variance in the BOLD signal explained by (A) both heart rate and respiration, (B) only heart rate, and (C) only respiration. In each panel, age group averages are shown for young and old participants. Voxels in which percent variance explained in younger adults was statistically significantly greater than older adults (*p* < 0.05 TFCE-corrected) are outlined in black, and alpha-fading was used to highlight sub-threshold voxels. For visualization purposes in the NKI dataset, *p* values were transformed using the natural logarithm to improve the contrast between highly significant voxels (e.g. *p* < 0.01). The brain slices shown are at z = -16 mm, 4 mm, 24 mm, 44 mm, and 64 mm in standard MNI152 space.

Results are presented in Figure 3. In both datasets, the percent variance in BOLD signal explained by heart rate and respiration was significantly greater in younger adults than in older adults, in the orbital frontal cortex (OFC), lateral ventricle, basal ganglia, and white matter (Fig. 3A). In the NKI dataset, percent variance of BOLD explained by heart rate and respiration was also significantly higher in the ACC and insula; these were also among the regions showing the strongest mean age-related differences in the HRV-ER dataset, though they did not survive the statistical threshold. The maps of percent variance of BOLD explained by heart rate (Fig. 3B) closely resembled the statistical maps resulting from jointly fitting heart rate and respiration (Fig. 3A). In both datasets, percent variance of BOLD explained by heart rate was higher in younger adults than in older adults in the lateral ventricles, basal ganglia, and white matter (Fig. 3B). There were also subthreshold differences in the HRV-ER dataset that were significant in the NKI dataset in the ACC, insula, and OFC. Interestingly, the percent variance in BOLD explained by respiration (e.g. RV in the NKI dataset and CO_2_ in the HRV-ER dataset) showed different age-related differences. In the NKI dataset, BOLD variance accounted for by RV was significantly higher in younger adults in OFC, insula, lateral ventricles, and ACC (Fig. 3C). In the HRV-ER dataset, older adults had slightly higher percent variance in BOLD accounted for by CO_2_ than younger adults in white matter and occipital cortex based on the group average maps; however, these differences were not statistically significant.

### 3.3 Age differences in the cross-correlation between the BOLD signal and physiological measures at different time lags

**Fig. 4.**
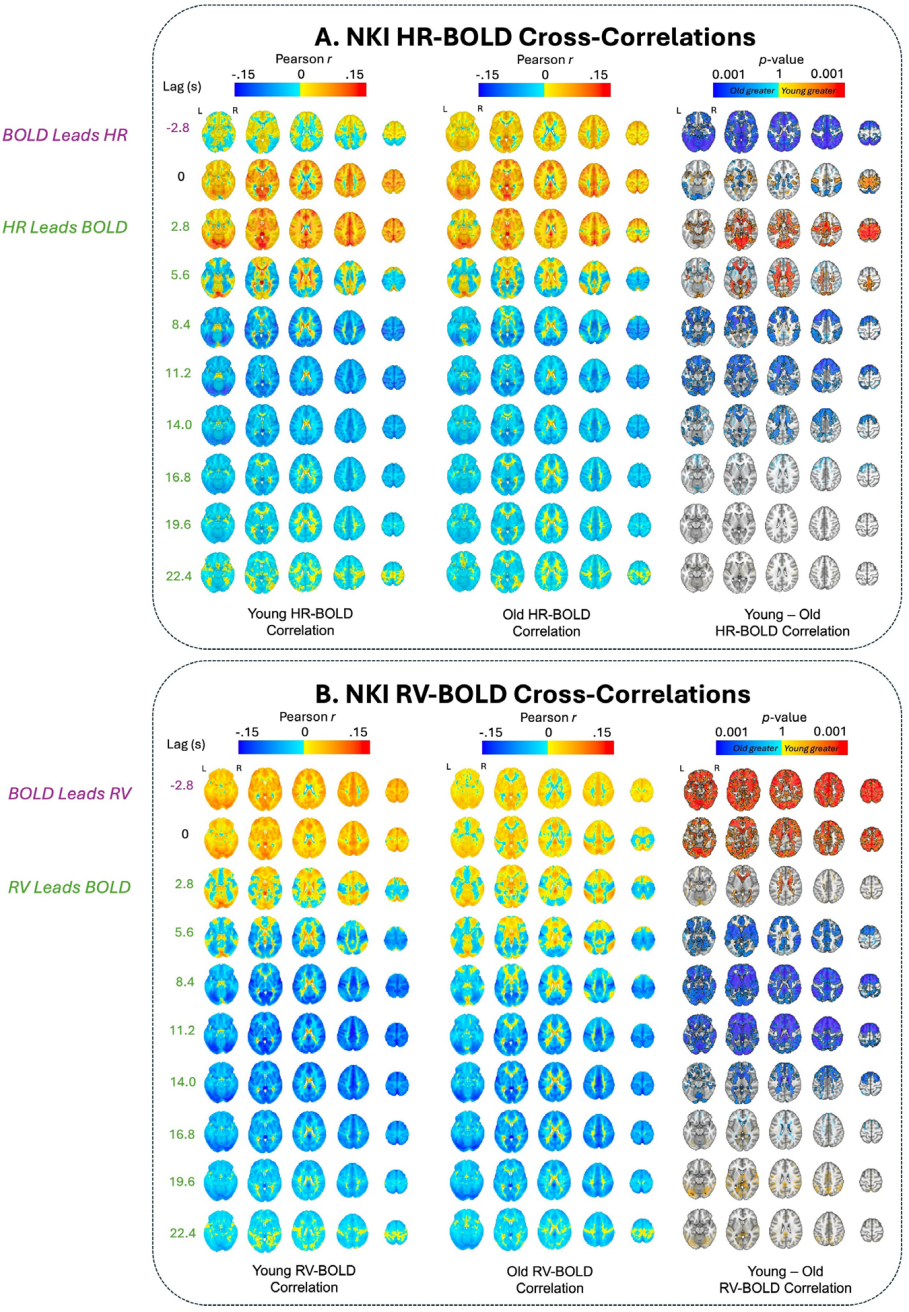
(A) HR–BOLD and (B) RV–BOLD cross-correlations for different time lags in the NKI dataset. Negative lags indicate that BOLD leads the physiological measure, and vice versa for positive lags. Age group averages for Pearson *r* coefficients are plotted at each lag. Significant voxels by age group at *p *< 0.05 (TFCE-corrected) are also outlined in black at each lag, along with alpha fading to show sub-threshold voxels. Red voxels indicate that young adult *r* values are greater than old adults, and blue voxels indicate that old adult *r* values are more positive (or less negative) than young adults. For visualization purposes, *p* values are transformed using the natural logarithm to improve the contrast between highly significant voxels (e.g. *p *< 0.01). The brain slices shown are at z = -6 mm, 4 mm, 24 mm, 44 mm, and 64 mm in standard MNI152 space.

**Fig. 5.**
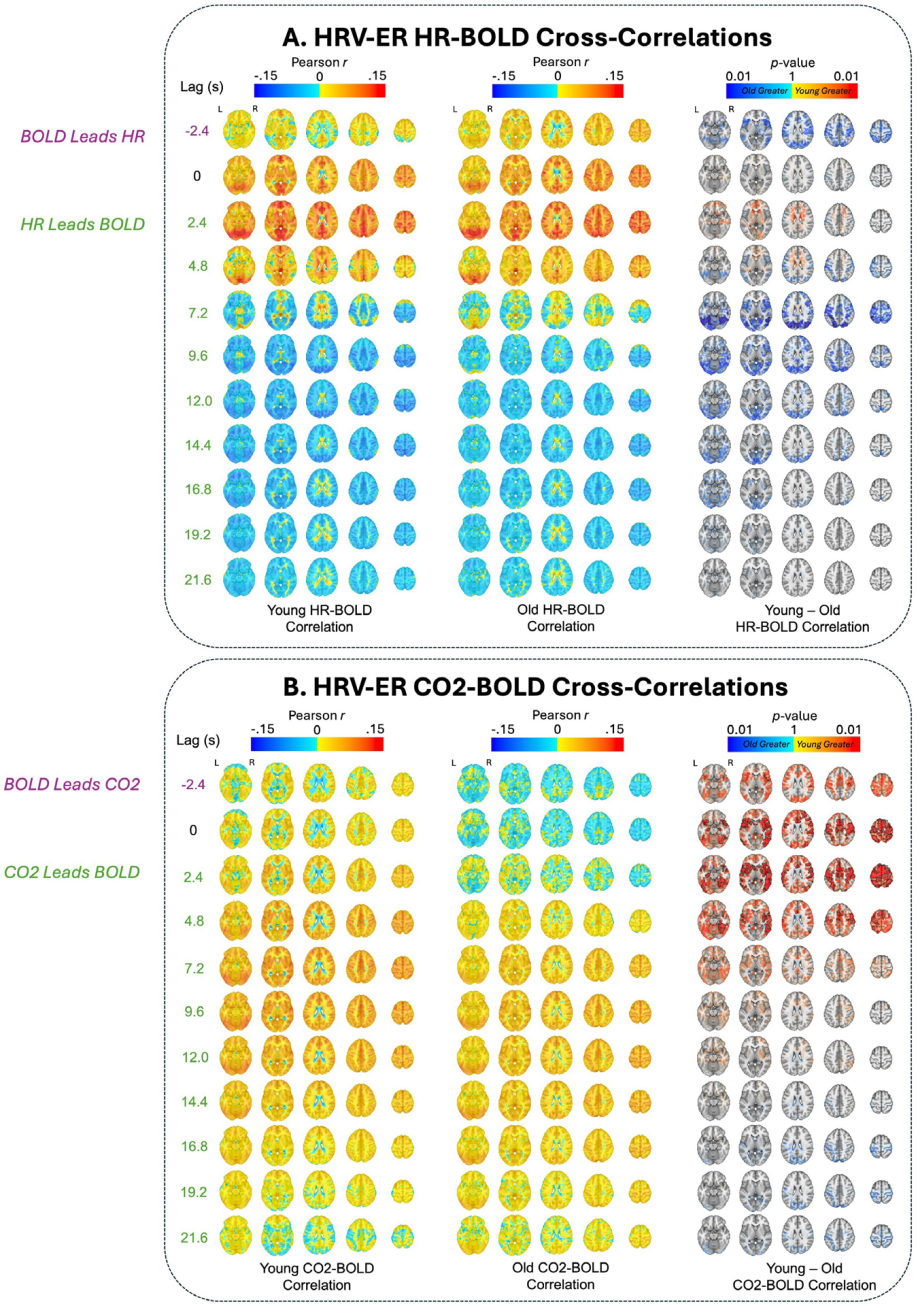
(A) HR–BOLD and (B) CO_2_–BOLD cross-correlations for different time lags in the HRV-ER dataset at baseline. Negative lags indicate that BOLD leads the physiological measure, and vice versa for positive lags. Age group averages for Pearson *r* coefficients are plotted at each lag. Significant voxels by age group at *p *< 0.05 (TFCE-corrected) are also outlined in black at each lag, along with alpha fading to show sub-threshold voxels. Red voxels indicate that young adult *r* values are greater than old adults, and blue voxels indicate that old adult *r* values are greater than young adults. The brain slices shown are at z = -16 mm, 4 mm, 24 mm, 44 mm, and 64 mm in standard MNI152 space.

To further examine how age impacts the dynamic association between physiological and BOLD signals, we computed the cross-correlations between the two signals (Section 2.5). In addition to revealing information about the temporal dynamics of BOLD–physiological coupling, a cross-correlation analysis does not assume specific hemodynamic impulse response models in the relationship between physiological and BOLD signals. Results for the NKI dataset are shown in Figure 4 and results for the HRV-ER dataset are shown in Figure 5, with significant clusters outlined in the figures (and discussed below) at TFCE-corrected thresholds of *p* < 0.05.

**Fig. 6.**
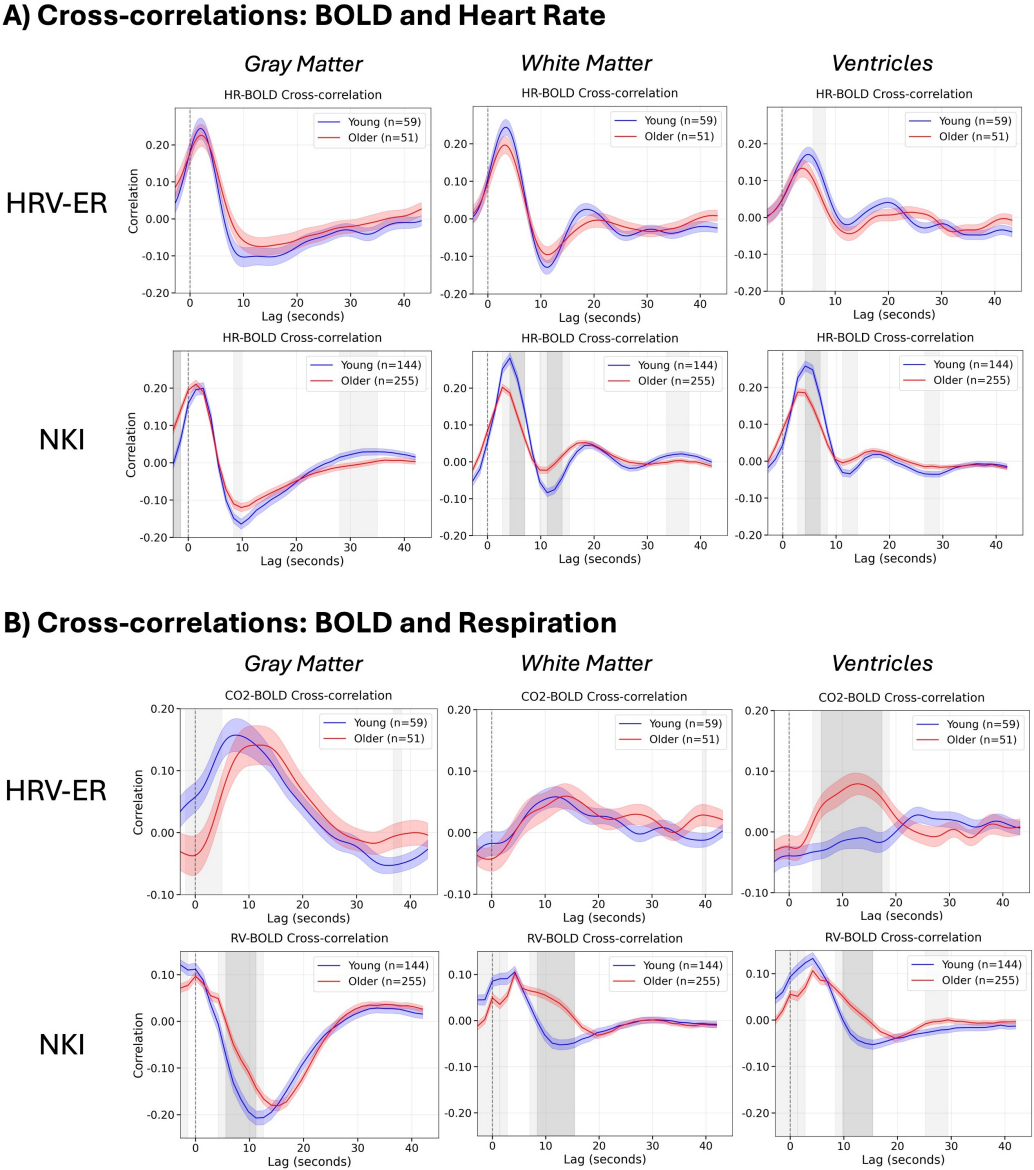
Cross-correlations between the BOLD signal and (A) heart rate and (B) respiration (i.e., CO_2_ for HRV-ER, RV for NKI), averaged across three tissue types: gray matter, white matter, and ventricles. In the HRV-ER data, both BOLD signal and CO_2_/HR are upsampled to TR = 0.2 seconds before cross-correlation calculation, but no upsampling occurred in the NKI data. Group averages for old and young adults are plotted along with shading for standard error. Lags where the cross-correlations between older and younger adults are statistically significant (*p *< 0.05) after Bonferroni correction are plotted in dark gray, and lags where the cross-correlation passed a *p *< 0.05 uncorrected threshold are plotted in light gray.

In the NKI dataset, the older adults exhibited significantly higher HR–BOLD cross-correlation across widespread regions at a lag of -2.8 seconds. In contrast, younger adults had significantly higher HR–BOLD cross-correlation in lags 2.8 seconds and 5.6 seconds in the ventricles, white matter, and ACC (Fig. 4A). In addition, younger adults had stronger negative HR–BOLD cross-correlations in the prefrontal cortex (PFC), OFC, and insula in lags 8.4 seconds and 11.2 seconds, as well as in the lateral ventricles in lags 11.2 seconds and 14.0 seconds (Fig. 4A). Younger adults also had more positive HR–BOLD cross-correlations at later time lags in the NKI dataset, between 30.8 seconds and 39.2 seconds, across the gray matter (Supplementary Fig. 3). Similar spatiotemporal trends were present in the HRV-ER dataset, albeit with smaller and/or nonsignificant effect sizes (Fig. 5). Here, the older adults also had significantly higher HR–BOLD cross-correlation across most of the brain at time lag of -2.4 seconds. The younger adults had higher subthreshold HR–BOLD cross-correlation in lags 2.4 and 4.8 seconds in a small number of voxels in the white matter, ventricles, ACC, and OFC (Fig. 5A). Younger adults also had significantly more negative HR–BOLD cross-correlation in occipital cortex at lag 7.2 seconds (Fig. 5A). In addition, younger adults had more negative sub-threshold differences in HR–BOLD correlations throughout the gray matter from lags 7.2 seconds to 19.2 seconds (Fig. 5A). These results are further supported by Figure 6A, which show that in the NKI dataset, HR–BOLD cross-correlations are significantly higher in younger adults at earlier lags (between 3 and 6 seconds) in white matter and ventricles, and significantly more negative in younger adults at later lags (between 10 and 15 seconds) across the whole brain. The HRV-ER dataset shows similar spatiotemporal trends with smaller and/or nonsignificant effect sizes (Fig. 6A).

In the NKI dataset, RV–BOLD cross-correlations were significantly higher in younger adults in the ventricles at lags -2.8 seconds, 0 seconds, and 2.8 seconds, as well as across the gray matter at lags -2.8 seconds and 0 seconds (Fig. 4B). From lags 5.6 seconds to 14 seconds, younger adults exhibited significantly more negative RV–BOLD cross-correlations in the ACC, basal ganglia, and PFC at lags 5.6, 8.4, and 11.2 seconds, with additional negative RV–BOLD correlations observed in the lateral ventricles at lags 8.4, 11.2, and 14 seconds (Fig. 4B). Similarly, in the HRV-ER dataset, younger adults exhibited significantly higher (*p* < 0.05 TFCE-corrected) CO_2_–BOLD cross-correlations in the OFC, insula, and ACC at lags 0, 2.4, and 4.8 seconds, with subthreshold differences appearing at lag -2.4 seconds and between 7.2 and 12 seconds (Fig. 5B). Figure 6B further illustrates this pattern, showing that in the NKI dataset, RV–BOLD cross-correlations were higher (*p* *<* 0.05 uncorrected) in younger adults than in older adults between 0 and 3 seconds in the white matter and ventricles. Additionally, younger adults exhibited significantly more negative RV–BOLD cross-correlations in all three tissue types at later lags between 7 and 12 seconds. This trend is consistent with the HRV-ER dataset, where CO_2_–BOLD cross-correlations in younger adults were greater in gray matter between -2 and 5 seconds (*p* *<* 0.05 uncorrected). These findings highlight an earlier onset of BOLD responsiveness to respiration in younger adults, as supported by both datasets.

### 3.4 Modulation of HR- and CO_2_–BOLD coupling by HRV biofeedback: Age and condition effects

**Fig. 7.**
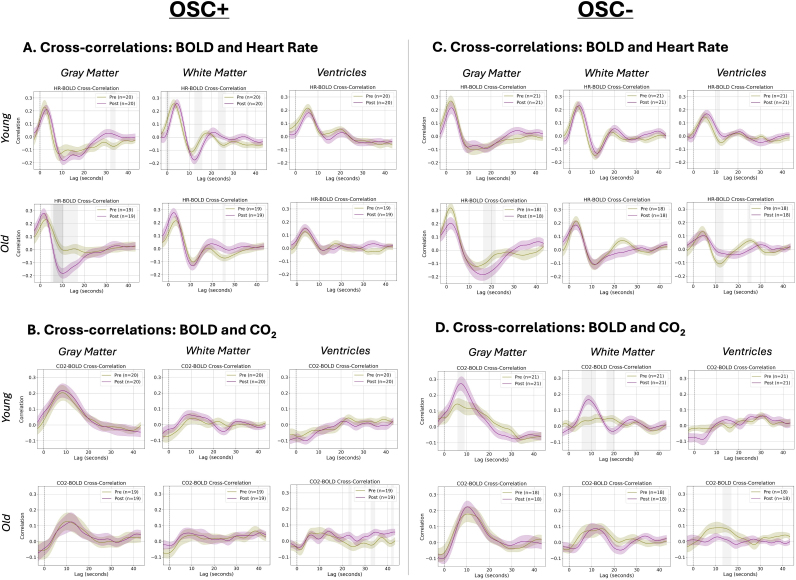
Impact of HRV biofeedback on BOLD–physiological cross-correlations. Cross-correlations between the BOLD signal (averaged across gray matter, white matter, and ventricles) and (A) HR and (B) CO_2_, before and after the Osc+ condition, are plotted. Cross-correlations between the BOLD signal (averaged across gray matter, white matter, and ventricles) and (C) HR and (D) CO_2_, before and after the Osc− condition, are plotted. Group averages are plotted along with standard error shading at every time point. Both BOLD signal and CO_2_/HR are unsampled to TR = 0.2 seconds before cross-correlation calculation. Lags where the cross-correlations between pre- and post-intervention are statistically significant (*p *< 0.05) after Bonferroni correction are plotted in dark gray, and lags where the difference passed a *p *< 0.05 uncorrected threshold are shown in light gray.

**Fig. 8.**
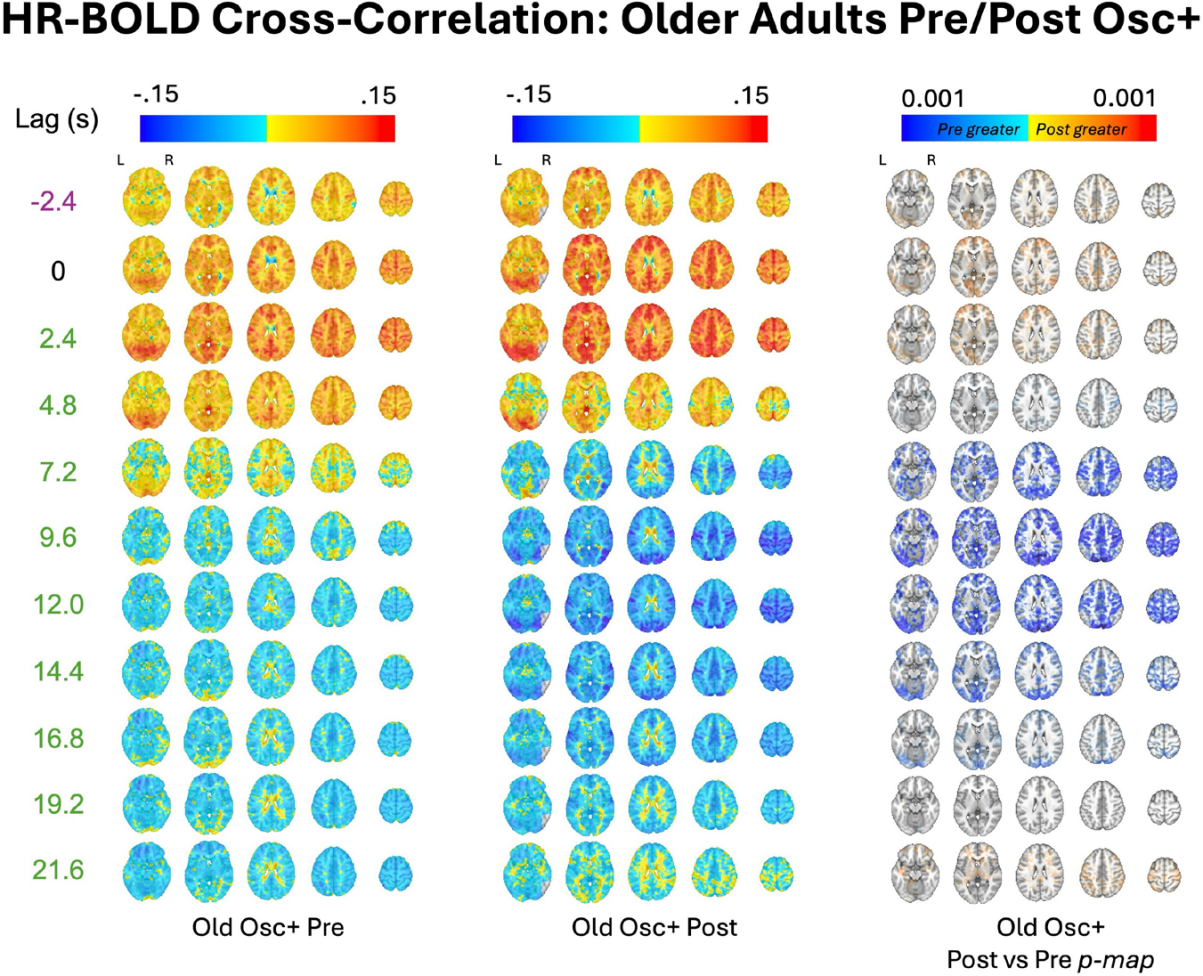
HR–BOLD whole-brain cross-correlation plots for older adults before and after the Osc+ intervention. Group averages for Pearson r coefficients are plotted at each lag. Significant voxels by age group at *p *< 0.05 (TFCE-corrected) are also outlined in black at each lag, along with alpha fading to show sub-threshold voxels. Red voxels indicate that post Osc intervention r values are greater than pre Osc intervention r values, and vice versa for blue voxels. The brain slices shown are at z = -16 mm, 4 mm, 24 mm, 44 mm, and 64 mm in standard MNI152 space.

To assess the effects of the HRV-biofeedback on physiological signal propagation into the fMRI BOLD signal, we computed whole-brain HR–BOLD and CO_2_–BOLD cross-correlations both before and after 5 weeks of each intervention separately for both age groups (Section 2.5). For older adults, the HR–BOLD cross-correlation was significantly more negative after the Osc+ intervention than before it (*p* < 0.05 Bonferroni-corrected) in the gray matter from 6 to 11 seconds, and this trend was present in the gray matter from 5 to 17 seconds (*p* < 0.05 uncorrected) (Fig. 7A). This effect is further evident in Figure 8, which shows the voxelwise equivalent of this whole-brain average score. Here, from 7.8 to 16.2 seconds, older adults had more negative HR–BOLD cross-correlations (subthreshold) throughout the gray matter after Osc+, and this result reached statistical significance between 9.6 and 12.0 seconds in the occipital cortex.

In addition, for participants in the Osc− condition, younger adults exhibited significantly greater (*p* *<* 0.05 TFCE-corrected) post–pre BOLD variance explained by HR and CO_2_ in the left temporal lobe (Supplementary Fig. 4A). This effect appears to be driven primarily by BOLD–CO_2_ coupling rather than BOLD–HR coupling, as younger adults had greater BOLD variance explained by CO_2_ in left OFC, left insula, left temporal cortex along with the lateral ventricles, bilateral occipital cortices, and bilateral PFC (Supplementary Fig. 3C). However, most of these voxel-level differences did not reach statistical significance after correcting for multiple comparisons using TFCE with 5000 permutations.

## 4 Discussion

### 4.1 General findings

This study examined the impact of aging on how heart rate and respiration modulate the fMRI BOLD signal across two independent datasets. Across both datasets, we observed significant age-related differences in HRV metrics, with younger participants demonstrating higher levels of RMSSD, HF HRV, and LF HRV than older participants. Younger adults also exhibited higher BOLD signal variance explained by heart rate and respiration across both datasets in areas such as the white matter, OFC, ventricles, insula, and ACC, results that were also mirrored in the cross-correlation analysis. In both the CO_2_–BOLD cross-correlations in the HRV-ER and the RV–BOLD cross-correlations in the NKI dataset, younger adults exhibited significantly earlier response onset than in older adults.

In the second dataset, we also examined whether several weeks of HRV biofeedback affected the relationships between BOLD signal and physiology. We found that HRV biofeedback training modulated physiological signal propagation into the BOLD signal in a condition- and age-dependent manner. Specifically, in older adults, the Osc+ condition caused HR-BOLD coupling to resemble a pattern more typical of younger adults. These findings offer new insights into the relationship between autonomic nervous system (ANS) function and brain aging, and we discuss their implications below.

### 4.2 HRV metrics and ANS regulation

The observed reduction in HRV metrics (ln RMSSD, ln LF, ln HF) in older adults aligns with prior research linking aging to diminished ANS regulation (Jandackova et al., 2016; Mather, 2024; Reardon & Malik, 1996; Thayer et al., 2021; Voss et al., 2012). HRV reflects the dynamic adaptability of the ANS to internal and external stimuli, encompassing both sympathetic and parasympathetic activity. Lower HRV, as observed in older adults, is indicative of reduced autonomic flexibility and cardiovascular adaptability, which have been associated with impaired baroreceptor sensitivity, vascular stiffening, and altered neurocardiac signaling pathways (Yugar et al., 2023; Ziegler, 2021). Consequently, cerebral blood flow becomes more susceptible to systemic blood pressure variations, potentially causing cerebral hypoperfusion or hyperperfusion (Ogoh & Tarumi, 2019). Over time, these disruptions may contribute to structural changes in the brain, including white matter lesions and microvascular damage, which are strongly linked to cognitive decline and neurodegenerative diseases such as Alzheimer’s (Badji et al., 2019; Han et al., 2021; Reeve et al., 2024).

### 4.3 Age-related differences in physiological–BOLD dynamics

To model the contribution of physiological signals to BOLD variance, we convolved basis functions for the cardiac response function (CRF) with HR (Chang et al., 2009; Chen et al., 2020) and for the respiratory response function (RRF) with RV (Birn et al., 2008; Chen et al., 2020) in the NKI dataset, while in the HRV-ER dataset, end-tidal CO_2_ was modeled with the end-tidal CO_2_ response function (Golestani et al., 2015). The peaks and troughs in these impulse response functions represent the characteristic temporal dynamics of physiological signal propagation into the BOLD signal. The use of flexible basis sets can capture differences in how fluctuations in HR, RV, and end-tidal CO_2_ drive BOLD signal changes across different regions and by age. Due to the potential non-uniqueness of explained variance in the cardiac-only or respiratory-only models, we focus our discussion on the age-related differences in BOLD variance explained by both cardiac and respiratory signals (Fig. 3A).

Regions such as the insula, OFC, ACC, and basal ganglia showed significant age differences in BOLD variance explained by physiological signals, which is noteworthy because these areas have been shown to exhibit reduced cerebral blood flow (CBF) with age (Lu et al., 2011). This suggests that diminished vascular reactivity or neurovascular coupling (linked with central autonomic activity) in these regions could contribute to the observed reductions in physiological–BOLD coupling in older adults. Although BOLD variance explained by heart rate and respiration showed global differences across the brain between younger and older adults in the NKI dataset, another reason for the regional specificity in age-related BOLD variance explained by physio differences seen in the HRV-ER dataset may be related to the central autonomic network (CAN). Beissner et al. (2013) showed prominent cortical hubs of the human CAN include the OFC, ACC, and bilateral insula—the same areas show the strongest age-related drop in BOLD variance explained by cardiac and respiratory regressors in our data. Given the age-related decline in autonomic tone, we interpret this spatial overlap as reflecting weakened neuronal engagement of the CAN. This may impair the concordance between neural activity in CAN hubs, ordinarily responsible for modulating cardiac and respiratory output, and the resulting peripheral physiological signals, thereby reducing the BOLD variance these signals explain in older adults. Future studies should probe how aging affects activity in the CAN using EEG or intracranial recordings to confirm whether age-related declines in neural engagement of the CAN drive reductions in autonomic tone.

Additionally, BOLD variance explained in white matter by physiological signals was significantly greater in younger adults. Modulation of vascular tone accompanying low-frequency fluctuations in systemic physiology may form a major component of white matter fMRI signals, particularly in periventricular white matter (Min et al., 2022; Özbay et al., 2018, 2019). The observed age-related reduction of white matter effects may align with previous studies implicating reduced structural integrity of white matter tracts due to arterial stiffening (Badji et al., 2019; Han et al., 2021). Arterial stiffening can compromise microvascular function, impairing oxygen delivery and potentially reducing the responsiveness of white matter to autonomic signals.

The finding of significant differences in the ventricles is also particularly intriguing. Younger adults showed greater fMRI signal variance explained by physiological signals in the ventricles, which might relate to cerebrospinal fluid (CSF) dynamics. Previous work suggests that negative cerebrovascular reactivity (CVR) in brain ventricles during fMRI reflects a dilation of ventricular vessels, decreasing the relative proportion of CSF per unit volume (Thomas et al., 2013). If these ventricular vessels are less responsive in older adults, it could explain the reduced variance in fMRI signals explained by physiological signals in these regions. Moreover, changes in breathing have been shown to directly alter both white matter and CSF signals through mechanisms such as sympathetically mediated effects on gray matter vascular tone (Picchioni et al., 2022). Our observations suggest that these global vascular dynamics may also be impaired in aging.

In the HRV-ER dataset, although older adults showed a slightly higher proportion of BOLD signal variance explained by CO_2_ in regions such as the occipital cortex—a somewhat counterintuitive pattern given other age-related differences—cross-correlation analyses also revealed that older adults had stronger BOLD–CO_2_ correlations in the ventricles at particular lags. By contrast, in the larger NKI dataset, younger adults typically demonstrated higher variance explained by RV throughout much of the brain. One possibility is that the Golestani et al. (2015) basis set for end-tidal CO_2_, derived from younger adults, may not fully capture older adults’ altered vascular responses. However, these findings may also suggest that mechanisms other than arterial CO_2_ concentrations—such as respiratory-correlated heart rate changes (De Meersman, 1993; Masi et al., 2007) and sympathetic nervous system activity on cerebrovasculature (Balasubramanian et al., 2019; Mather, 2024; Rim et al., 2022)—could drive age differences in respiration-related brain hemodynamics in the NKI dataset. Because RV and CO_2_ were collected in separate datasets with different sample sizes, it remains unclear whether the observed discrepancies reflect genuine physiological differences or are partly influenced by sampling variation, reduced power, or different measurement modalities (RV vs. CO_2_). Future studies that measure RV and CO_2_ concurrently will be essential to replicate and further investigate these effects.

While both RV and end-tidal CO_2_ relate to the arterial CO_2_ changes that modulate cerebral blood flow, they differ in temporal dynamics, temporal resolution, and physiological sources: RV reflects instantaneous changes in tidal volume and breathing rate, whereas end-tidal CO_2_ samples alveolar CO_2_ only during exhalation and does not capture respiratory effects at higher temporal resolutions. Although some of the physiological mechanisms driving RV and PETCO_2_ are shared, they still contribute distinct information about physiological information. This is an important point to consider when interpreting the results of our study, especially the differences in RV and CO_2_ fluctuations in BOLD activity.

It is important to note that the PVE by physiological signals in the BOLD signal represents a fractional, not absolute, contribution to BOLD variance. Thus, an observed decrease in PVE for older adults does not necessarily reflect the raw amplitude of physiological responses alone; instead, it can arise from changes in both the numerator (physiologically and neuronally driven variance) and the denominator (total BOLD variance). Therefore, another potential interpretation of our findings is that higher levels of resting-state BOLD variance could account for lower PVE by physiological signals in the BOLD signal in older adults. However, this scenario is unlikely as it is well documented that resting-state BOLD variance declines with age (Garrett et al., 2010; Kumral et al., 2020; Millar et al., 2020; Tsvetanov et al., 2021). Alternatively, if neuronal variability increases in older adults or if neuronal stimuli generate proportionally more BOLD variance than physiological fluctuations, the fraction of variance explained by physiological signals could appear lower—even if absolute physiological responses remain unchanged. Yet empirical evidence generally suggests that aging diminishes neuronal hemodynamic responses of the brain (Fabiani et al., 2014; Ward et al., 2015; West et al., 2019). For example, older adults show reduced hemodynamic response function amplitude during a visual–auditory task (West et al., 2019). In sum, the most likely explanation for our observed decrease in physiological PVE with age is that vascular and structural changes attenuate the absolute BOLD response to physiological fluctuations (Badji et al., 2019; Han et al., 2021; Hoiland et al., 2019; Lu et al., 2011; Reeve et al., 2024; Rucka et al., 2015). Future studies that simultaneously measure neuronal and physiological components of BOLD variability, along with direct indices of vessel compliance or vascular tone, will help clarify these mechanisms.

### 4.4 Time lag dynamics and cerebrovascular response

In both younger and older adults, the HR–BOLD and RV–BOLD cross-correlations demonstrated canonical patterns, resembling the shapes of the cardiac response function (CRF) and respiratory response function (RRF), respectively. These patterns are characterized by an initial positive peak followed by a decline, transitioning to negative correlations at later lags (Supplementary Fig. 2). The temporal profiles and regional distributions of these correlations align with findings from previous studies (Birn et al., 2008; Chen et al., 2020; Gold et al., 2024), which similarly reported early positive correlations transitioning to negative correlations as a hallmark of BOLD–physiological coupling. Across both NKI and HRV-ER datasets, HR–BOLD cross-correlation was stronger in younger adults in areas that are highly vascularized, including the orbitofrontal cortex (OFC), basal ganglia, ventricles, and white matter tracts supplied by major arteries such as the anterior cerebral artery (ACA), middle cerebral artery (MCA), and posterior cerebral artery (PCA). While age-group differences in the HRV-ER dataset did not survive TFCE correction, this dataset showed similar trends as those in the NKI dataset. Since the HRV-ER dataset has a much smaller sample size, it is possible that, with more statistical power, these differences would have reached significance. Overall, our HR–BOLD cross-correlation results provide additional support for the stronger BOLD variance explained by heart rate in younger adults.

Of note, a recent study also examined cross-correlation between physiological signals and BOLD signals in the Human Connectome Project Aging (HCP-A) dataset (Fan et al., 2025). In contrast to our results, this study found that the oldest adults (60+ years) in the HCP-A cohort exhibited larger peak cross-correlation between inter-heart-beat intervals (HBIs, i.e., the inverse of heart rate) and global cortical BOLD signal than middle-aged (36–60 years) and younger (18–36 years) adults. Importantly, however, the oldest adults in this HCP-A cohort had significantly higher RMSSD HRV than the middle-aged group. Younger adults in our datasets, in contrast, had higher RMSSD than older adults (although non-significant for the NKI dataset). This raises an important question about whether baseline physiology may underlie HR–BOLD cross-correlations that is to be explored in future studies.

The CO_2_–BOLD correlations in our study provide further insight into the role of cerebrovascular response and vascular propagation in age-related changes in neurovascular dynamics. First, Figure 6 reveals a notable consistency between the CO_2_–BOLD responses in the HRV-ER dataset and the (negative deflection of the) RV–BOLD responses in the NKI data, a dynamic relationship that may be expected from prior reports with concurrent RV and end-tidal CO_2_ monitoring (Chang & Glover, 2009). In the HRV-ER dataset, younger adults exhibited faster CO_2_–BOLD cross-correlation peaks than older adults, which could translate to a more rapid cerebrovascular response to changes in metabolic demands (Hoiland et al., 2019; Lu et al., 2011). Similarly, in the NKI dataset, younger adults showed an earlier onset of the dip in RV–BOLD cross-correlations, which may mirror this faster cerebrovascular response, suggesting a consistent age-related pattern across datasets. Fan et al. (2025) also studied cross-correlations between RV and global cortical BOLD in the HCP-A dataset and found that dip in RV–BOLD cross-correlation occurs later in older adults than in younger adults, which is consistent with our findings. Cerebrovascular response, defined as the ability of blood vessels to dilate in reaction to increases in CO_2_, is crucial for maintaining oxygen delivery and cerebral perfusion. In younger adults, this response is more synchronized and efficient, leading to quicker adjustments in blood flow (Hoiland et al., 2019; Lu et al., 2011). In contrast, older adults exhibited delayed CO_2_–BOLD correlation peaks, which may reflect a slower cerebrovascular response and cerebral blood flow due to age-related arterial stiffening and reduced vessel compliance (Badji et al., 2019; Hoiland et al., 2019; Lu et al., 2011; Reeve et al., 2024; Rucka et al., 2015). These changes impair the dynamic ability of the vascular system to transmit blood flow signals efficiently, delaying the response to CO_2_ fluctuations.

### 4.5 HRV biofeedback and physiological modulation

Our findings regarding HRV biofeedback highlight intriguing age- and condition-dependent effects. In the HRV-ER dataset, the Osc+ intervention (which aimed to enhance HR oscillations) in older adults resulted in more negative HR–BOLD cross-correlations at the lags between 6 and 11 seconds, closely resembling the patterns typically observed in younger adults. This suggests that increasing HR oscillations through biofeedback may partially restore age-related declines in the cerebrovascular response. Such a shift toward a more “youth-like” physiological response profile may reflect enhanced autonomic flexibility and improved vascular responsiveness (Deschodt-Arsac et al., 2018; Fournié et al., 2021; Mohapatra et al., 2024)—perhaps even with implications for neural activity (Bright et al., 2020; Mather &Thayer, 2018).

Indeed, as proposed by Mather and Thayer (2018), high-amplitude oscillations in heart rate may enhance functional connectivity within emotion–regulation networks—especially in medial prefrontal areas sensitive to physiologically driven oscillatory input. Additional evidence of the potential neural benefits of higher-amplitude heart rate oscillations in older adults comes from Jung et al. (2024), who found that changes in resting HRV significantly mediated reductions in negative emotion in the group instructed to increase HR oscillations (Osc+). Thus, when older adults successfully enhanced their cardiac dynamics, they also experienced improved emotional well-being—an effect directly tied to changes in autonomic functioning (Mather & Thayer, 2018). Our observations are also in line with recent work examining other aspects of the HRV-ER dataset, which demonstrate multiple benefits associated with increasing HR oscillations. For instance, Yoo et al. (2022) reported that daily practice to augment HR oscillations (Osc+) led to increased cortical volume in the left orbitofrontal cortex (OFC) for both younger and older adults, suggesting a positive impact on key prefrontal regulatory regions. Beyond emotion regulation, Min et al. (2023) show that Osc+ training decreased plasma Alzheimer’s disease (AD)-related biomarkers (plasma Aβ40 and Aβ42), whereas the Osc− condition actually increased these biomarkers. This suggests a link between autonomic regulation and pathways implicated in AD pathophysiology.

Taken together, these convergent findings demonstrate that interventions designed to boost heart rate oscillations can partially restore age-related declines in autonomic and vascular responsiveness. Our results also suggest that HR–BOLD coupling may serve as a biomarker of autonomic health in older adults, aligning with broader evidence that links increasing vagal tone to enhanced emotional well-being and potentially to neuroprotective benefits.

### 4.6 Role of physiological state

Interestingly, the differences in magnitude of HR–BOLD correlations between older and younger adults (Figs. 4, 5) suggest potential parallels with the vigilance-related fMRI-autonomic covariance patterns observed by Gold et al. (2024). This study highlighted that fMRI-autonomic covariance differs across baseline vigilance states, with stronger variance in fMRI explained by physiological signals during low vigilance, which is associated with reduced arousal and parasympathetic dominance. In contrast, high vigilance states, characterized by heightened norepinephrine levels and sympathetic dominance, were associated with weaker fMRI–autonomic coupling. Older adults in our study, with their observed patterns of delayed HR–BOLD and RV–BOLD correlations, may resemble this higher vigilance state. Sympathetic dominance in older adults (Mather, 2024) could explain these temporal differences—particularly as the older-adult cohorts in our study exhibited reductions in high-frequency heart rate variability and RMSSD, compared with the younger adults.

In addition to the analysis described in previous sections, we sought to clarify whether the age-related changes in BOLD–physiological differences were arising due to age-induced changes in the coupling of physiological signals to the BOLD signal, or just age-related changes in physiological signals themselves (Jandackova et al., 2016; Reardon & Malik, 1996; Voss et al., 2012). There is also evidence of sex differences in HRV (Koenig & Thayer, 2016) as well as sex differences in vascular aging (Ji et al., 2022), which may have influenced our results. To isolate the effect of aging on BOLD–physiological coupling as the underlying mechanism explaining our findings, we replicated our PVE and cross-correlation analysis while controlling for variables such as sex, average heart rate, LF HRV, HF HRV, breathing rate, average end-tidal CO_2_ (for the HRV-ER dataset), and standard deviation of RV (in the NKI dataset). Across both datasets, we observed that controlling for these covariates had no effect on the age-related differences in BOLD–HR, BOLD–RV, or BOLD–CO_2_ cross-correlations (Supplementary Fig. 5). Additionally, in the HRV-ER dataset, adjusting for average HR, LF HRV, and HF HRV had no impact on the Osc+ mediated difference in HR–BOLD cross-correlation among older adults (Supplementary Fig. 6).

In the HRV-ER dataset, controlling for gender, LF and HF HRV, average HR, and average end-tidal CO_2_ increases the number of voxels in which younger adults’ BOLD PVE by HR and end-tidal CO_2_ is significantly greater than older adults’ BOLD PVE, particularly in gray matter regions, such as the orbitofrontal cortex, anterior cingulate cortex, and insula (Supplementary Fig. 7). However, additionally controlling for breathing rate removes all significant voxels (Supplementary Fig. 7), suggesting that breathing rate is tightly linked to the observed coupling differences in this cohort. In the NKI dataset, controlling for gender, average HR, LF and HF HRV, standard deviation RV, and breathing rate seemed to preserve most of the significant age-related differences in BOLD PVE by HR and RV in the gray matter and periventricular white matter, although there appears to a reduction in significant voxels in white matter (Supplementary Fig. 8). This seems to suggest that some of the white matter PVE differences outside of the periventricular area may be attributable to group differences in baseline physiology, whereas the core age effect in the gray matter reflects genuine alterations in physiological–BOLD coupling. Taken together, these findings underscore the complexity of interpreting physiological–BOLD coupling in aging research, as different physiological covariates can either reveal or mask significant group differences.

### 4.7 Implications for physiological preprocessing and data collection in fMRI studies

Our finding that younger adults exhibit markedly stronger BOLD coupling to heart rate and respiration fluctuations than older adults raises practical questions for routine fMRI preprocessing. For example, if one wishes to remove physiological effects from fMRI data, should such “corrections” be applied differently in younger versus older populations? Further, if physiological effects provide valuable insight into the aging brain, in which cases should they be removed?

Importantly, current tools do not readily discern whether physiological influences on the BOLD signal in a given region reflect changes in neuronal activity, vascular effects, or both. Simply regressing out a subspace associated with peripheral physiology would remove neuronal and vascular effects together, including cognitive or emotional processes closely linked with autonomic modulation. Therefore, future studies need to examine in more depth the specific mechanisms driving the physiological component of the BOLD signal, and whether the respective contributions of these mechanisms differ in older versus younger adults. A clearer understanding of these mechanisms would also help researchers to handle physiological effects in a manner that is optimized to the specific goals of the study. For example, cognitive neuroscience studies aiming to identify specific task-evoked neural activity may prefer to preserve as much true neural signal as possible, even if some (vascular) physiological effects remain uncorrected. Conversely, studies focused on cerebrovascular function may want to retain vascular contributions to the BOLD signal, as these are of direct interest, while minimizing confounding neural fluctuations.

We can, however, strongly recommend future aging-related fMRI studies to collect concurrent measures of cardiac and respiratory activity in both older and younger adults, especially since the extent to which these signals modulate the BOLD signal is different with age. These data provide a critical reference point for understanding how physiological processes modulate the BOLD signal in different brain regions and age cohorts. Without such recordings, researchers may inadvertently attribute group differences in BOLD signal to cognitive or neural factors when, in fact, they may stem from group differences in systemic physiology or brain–body dynamics. Thus, concurrent recordings may improve interpretability and allow researchers to better contextualize their findings.

### 4.8 Limitations and future directions

Several limitations should be noted. First, the canonical impulse responses used to model the BOLD response to heart rate, respiration, and CO_2_ were originally derived from younger adults. It is possible that these impulse responses do not fully capture the altered vascular dynamics characteristic of older adults, potentially underestimating the percent variance explained in this group. While our model-free cross-correlation analyses largely corroborate the presence of age-related differences, developing age-specific physiological response functions would be a valuable direction for future work. Second, the temporal resolution for all of our cross-correlation analyses is inherently limited by the image TR, and future studies with shorter TR may be able to better capture the temporal dynamics of physiological–BOLD coupling. Third, the relatively small sample size for post-intervention scans in the HRV-ER dataset limits statistical power, making it more challenging to detect subtle effects and interactions. Fourth, participants’ adherence to the daily HRV biofeedback protocols may have varied, and differences in training engagement or compliance could influence the observed intervention effects. Fifth, structural differences between younger and older adult brains (e.g., age-related atrophy or enlarged ventricles) may not be fully accounted for by standard preprocessing pipelines, potentially introducing systematic biases in image registration and alignment. Future work could use age-specific templates or advanced normalization methods to better accommodate these structural differences. Sixth, as we focused here on older adults in the range of 50–85 years old, it is possible that the specific age range may impact the results of old versus young group comparisons. Further, differences in imaging parameters and preprocessing pipelines could introduce technical variability in characterizing relationships between fMRI and peripheral physiological signals. Future studies employing multi-modal imaging (e.g., near-infrared spectroscopy or arterial spin labeling) and longitudinal follow-ups could provide a more comprehensive view of how autonomic regulation interventions influence cerebrovascular function and healthy brain aging over time. Finally, for accurate HRV quantification (Section 3.1), higher sampling rates for PPG recordings (e.g., ≥250 Hz) can help to capture the fine temporal resolution needed for precise inter-beat interval detection (Kwon et al., 2018). While the NKI physiological sampling rate was only 62.5 Hz, the HRV results that we find in the NKI data appear to follow an expected age-related trend and align well with the results of the HRV-ER data, which had a higher PPG sampling rate.

## Ethics

For the HRV-ER dataset, The University of Southern California Institutional Review Board approved the study. All participants provided written, informed consent prior to participation and received monetary compensation for their participation. The NKI dataset was openly provided and the study was approved by the institutional review board at the NKI.

## Data and Code Availability

All source code and instructions for replicating the results in this paper can be found at: https://github.com/richardwsong/physio-bold-aging

Preprocessed physiological and fMRI data for the HRV-ER dataset are accessible here: https://vanderbilt.box.com/s/ff388e0q9yzx2qkab273q01p9ieo2c2a

The Enhanced Nathan Kline Institute - Rockland Sample dataset is publicly available at the following repository: http://fcon_1000.projects.nitrc.org/indi/enhanced/. The HRV-ER dataset is publicly available at https://openneuro.org/datasets/ds003823.

## Author Contributions

R.S.: Data preprocessing, Conceptualization, Formal analysis, Methodology, Visualization, Writing—original draft, Writing—review and editing. J.M.: Data curation, Writing—Review and editing, Methodology, Validation. S.W., K.R.O., S.E.G.: Data preprocessing, Writing—review and editing, Methodology. R.Y.: Data preprocessing. H.J.Y., K.N.: Data curation. J.E.C.: Investigation, Writing—review and editing, Methodology. M.M., C.C.: Conceptualization, Investigation, Methodology, Writing—original draft, Writing—review and editing, Project administration, Funding acquisition

## Funding

This work was supported by NIH grants RF1MH125931 (C.C., M.M.), F99AG079810 (S.E.G.), T32 MH064913 (K.R.O.), and R00NS118120 (J.E.C.), and by the Sally and Dave Hopkins Faculty Fellowship (C.C.).

## Declaration of Competing Interest

The authors have no conflicts of interest.

## Supplementary Materials

Supplementary material for this article is available with the online version here: https://doi.org/10.1162/IMAG.a.99

## Supplementary Material

Supplementary Material
