# Supplementary material for: The physiological component of the BOLD signal: Impact of age and heart rate variability biofeedback training
Source: Imaging Neurosci (Camb). 2025 Aug 7;3:IMAG.a.99. doi: 10.1162/IMAG.a.99 (PMC12336062; doi:10.1162/IMAG.a.99)
Supplement: Supplementary Material [file IMAG.a.99_supp.pdf]

Supplementary Material

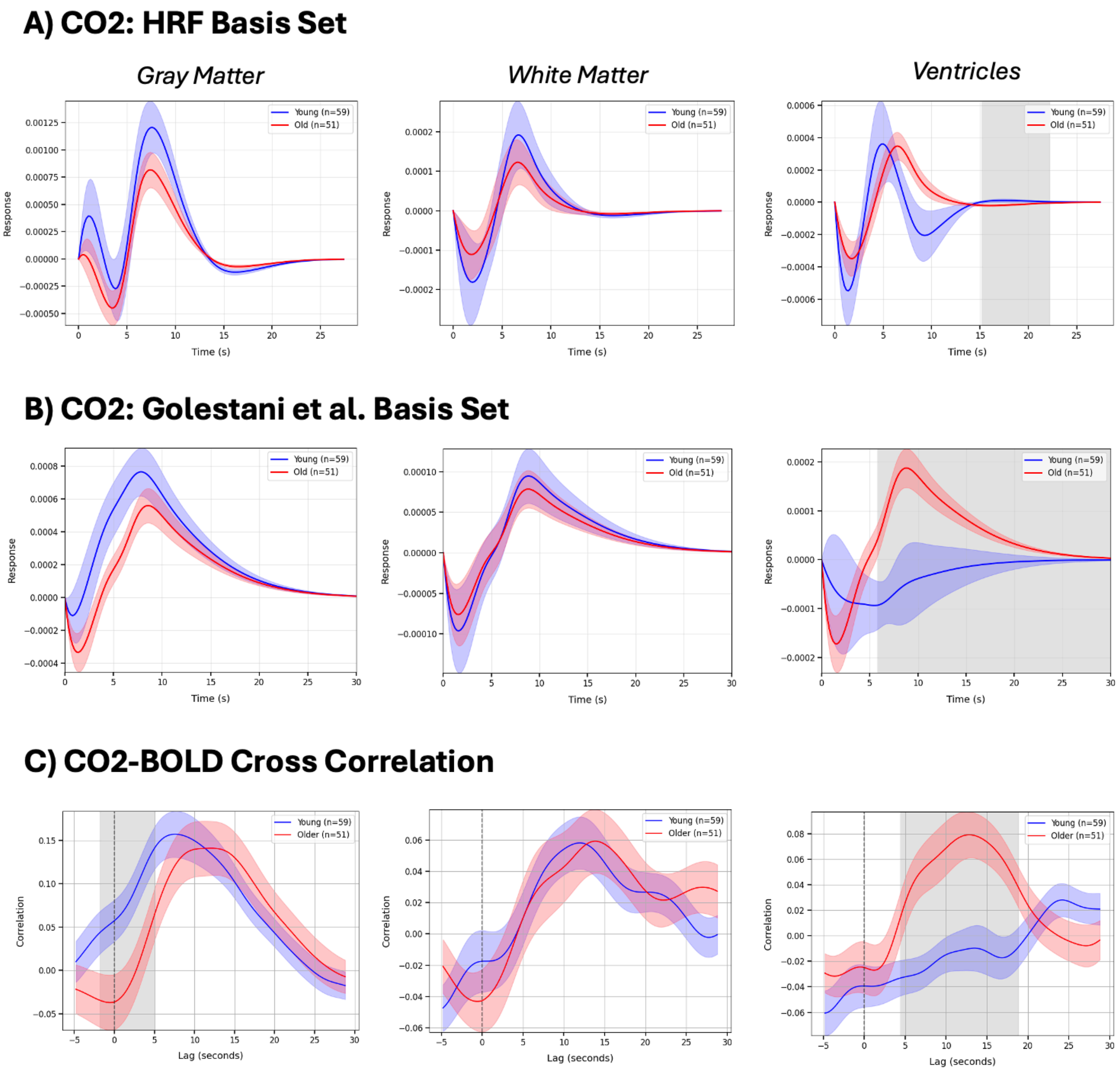

Supplemental Figure 1. Modeling end-tidal CO2 response with 2 candidate basis sets A) Canonical HRF and B) Golestani et al. (2015) Basis sets were derived by taking a weighted sum of the respective beta values with each basis function after least squares fitting between convolved end-tidal CO_2 regressors and the BOLD signal averaged across gray matter, white matter, or ventricles. In the HRV-ER data, both CO_2 and BOLD signals were unsampled to TR = 0.2 seconds before least squares fitting. C) CO_2-BOLD cross-correlation is shown for reference. Lags/time-points where the response or correlation is statistically significant (p<0.05) between older and younger adults are shaded in gray.

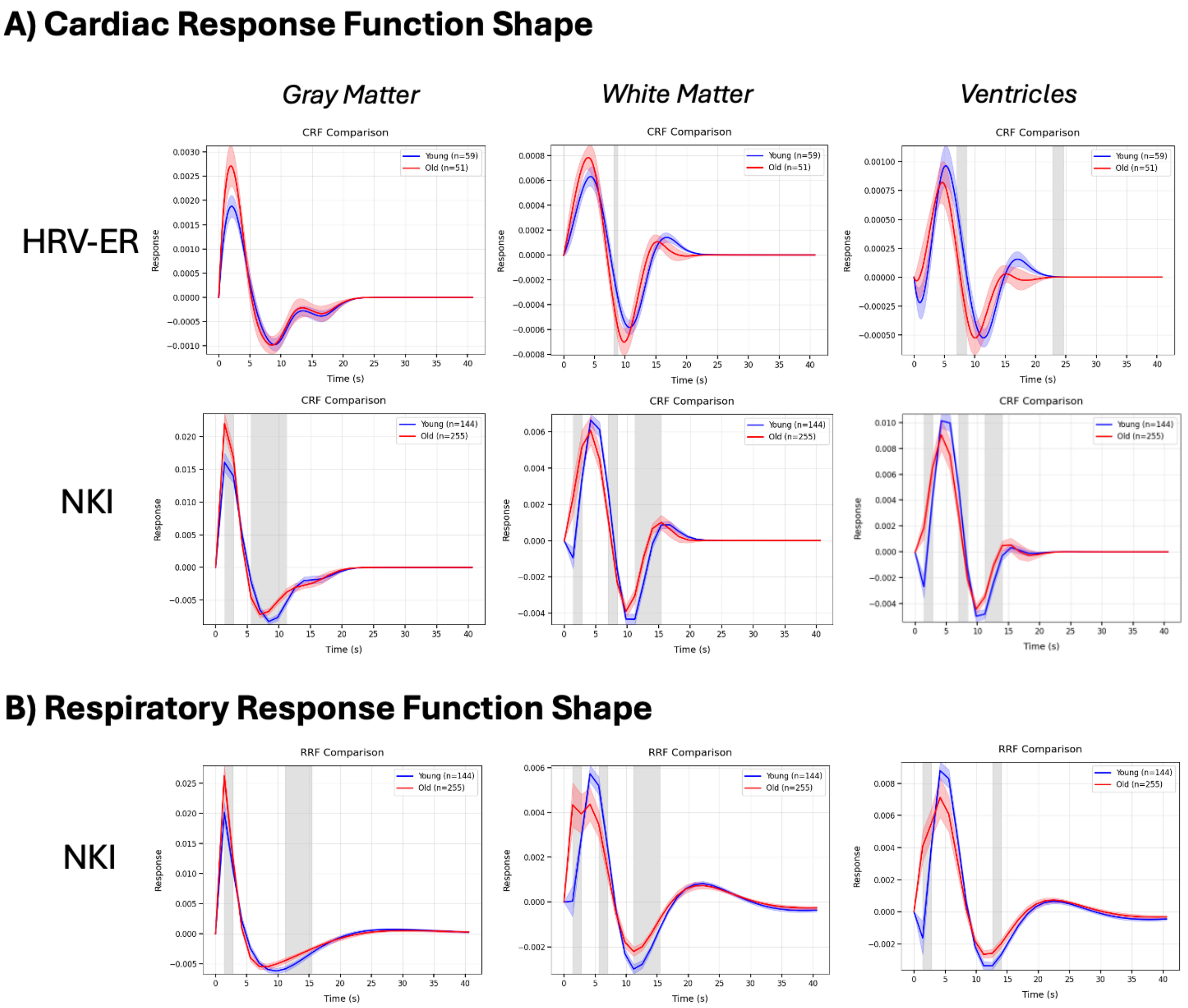

Supplemental Figure 2. Modeling HR- and RV- induced BOLD response using A) Cardiac Response Function (CRF) and Respiratory Response Function (RRF). Basis sets were derived by taking a weighted sum of the respective beta values with each basis function after least squares fitting between convolved HR/RV regressors and the BOLD signal averaged across gray matter, white matter, or ventricles. In the HRV-ER data, both HR and BOLD signals were unsampled to TR = 0.2 seconds before least squares fitting. Time-points where the response or correlation is statistically significant ($p<0.05$) between older and younger adults are shaded in gray.

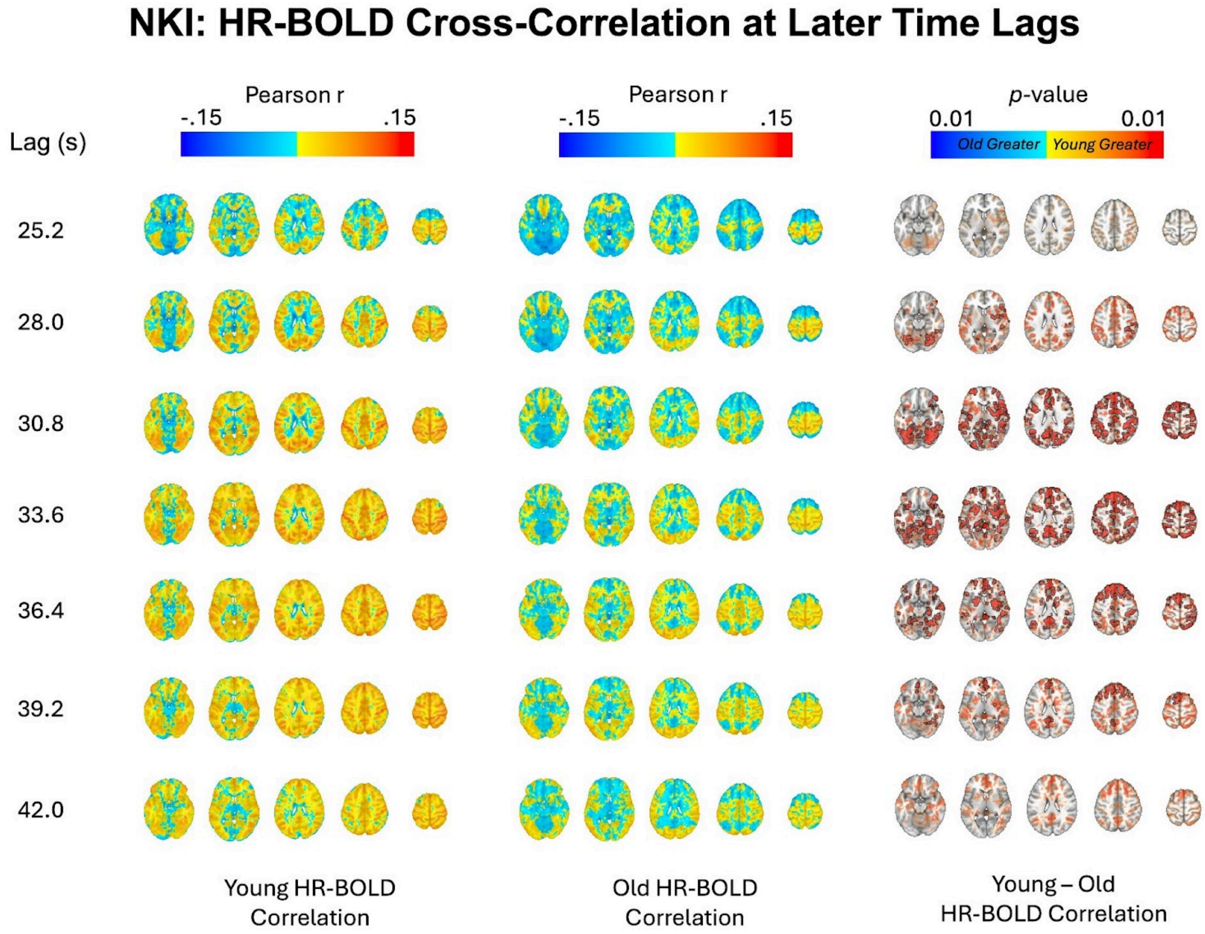

Supplemental Figure 3. HR-BOLD cross-correlations at lags 25.2 through 42.0 seconds in the NKI dataset. Age group averages for Pearson *r* coefficients are plotted at each lag. Significant voxels by age group at $p < 0.05$ (TFCE-corrected) are also outlined in black at each lag, along with alpha fading to show sub-threshold voxels. Red voxels indicate that young adult *r* values are greater than old adults. The brain slices shown are at z = -16 mm, 4 mm, 24 mm, 44 mm, and 64 mm in standard MNI152 space.

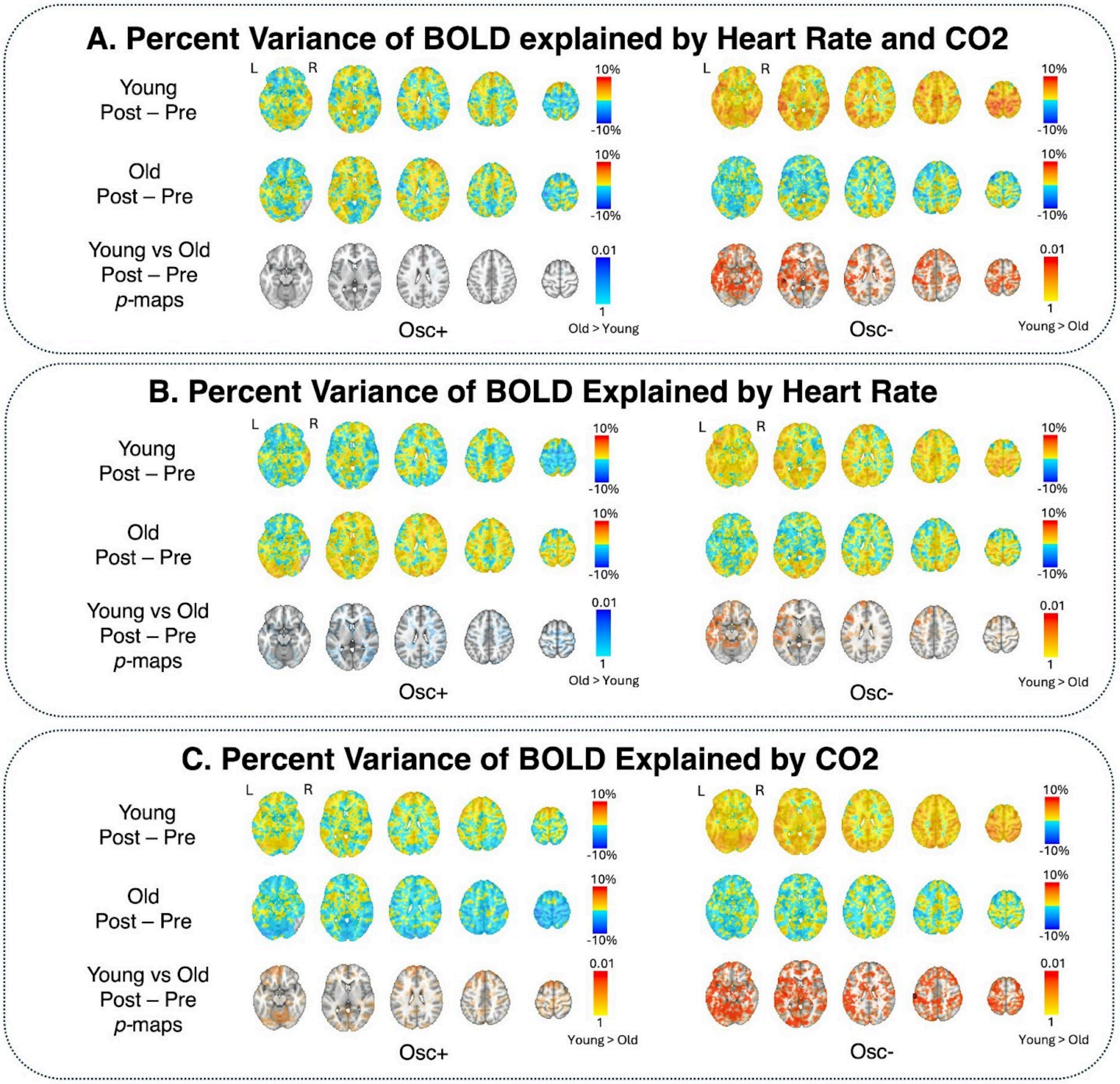

Supplemental Figure 4. Difference in percent variance of BOLD explained by A) HR and CO2, B) HR, and C) CO2 between pre and post 5 week HRV-biofeedback intervention in the HRV-ER dataset. In each panel, group averages for young adults and older adults are shown in the first two rows, and p-maps comparing the two age groups are shown in the third row. Significant voxels at $p < 0.05$ (TFCE-corrected) are outlined in black, and alpha-fading was used to depict subthreshold voxels.

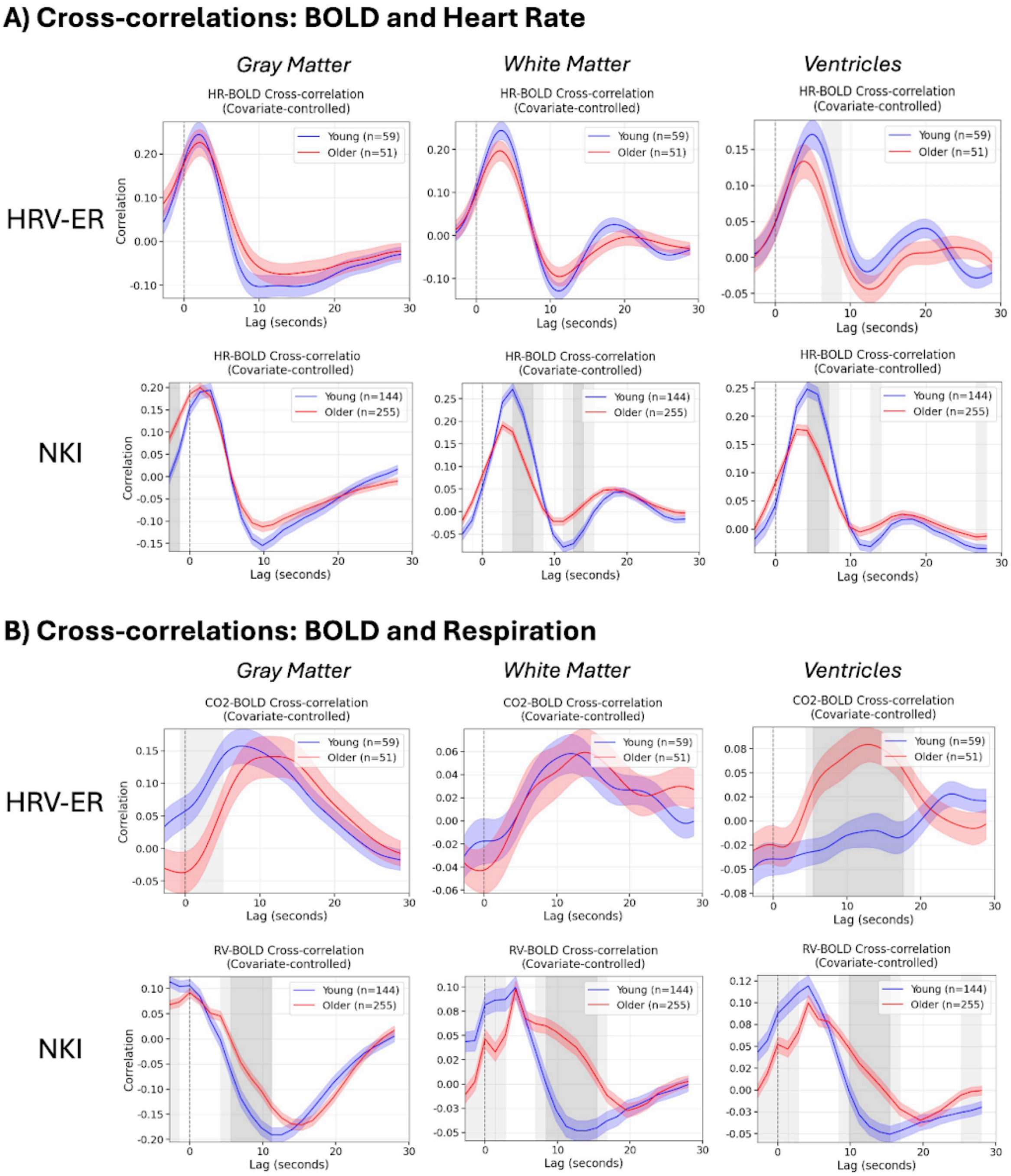

Supplemental Figure 5. Cross-correlations between the BOLD signal and A) heart rate and B) respiration (i.e., CO_2 for HRV-ER, RV for NKI), averaged across three tissue types: gray matter, white matter, and ventricles. Lags where the cross correlations between older and younger adults were statistically significant (p<0.05) after Bonferroni correction are plotted in dark gray, and lags where the cross correlation passed a p<0.05 uncorrected threshold are plotted in light gray. HR-BOLD cross-correlation significance tests were corrected for average heart rate, LF HRV, HF HRV, and RMSSD. CO_2-BOLD cross-correlations were corrected for average CO_2 and breathing rate, and BOLD-RV cross correlations were corrected for average RV and breathing rate.

HRV-ER: Gray Matter (Older Adults Osc+ Pre vs Post)

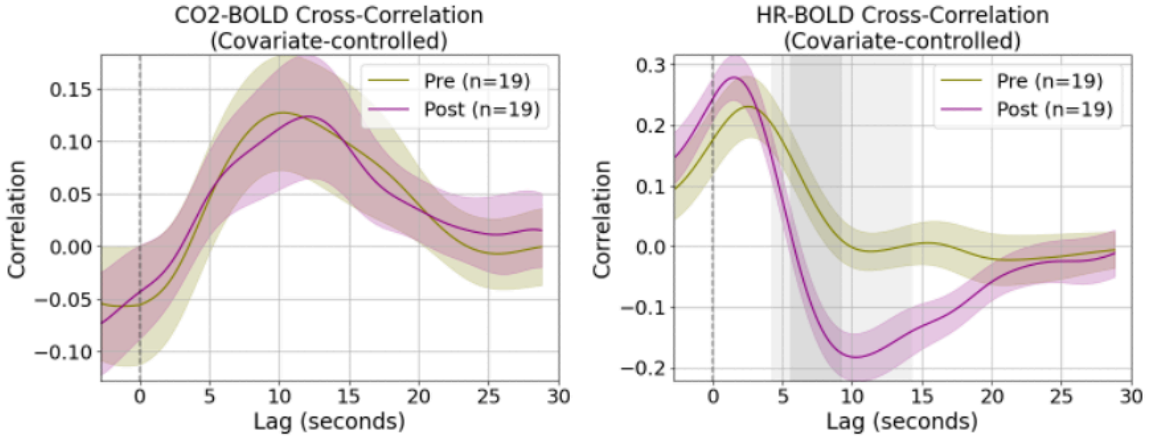

Supplemental Figure 6: A) Cross-correlations between CO2 and HR with BOLD signal in the gray matter for older adults before and after the Osc+ intervention. Lags where the cross correlations between pre and post intervention were statistically significant ($p<0.05$) after Bonferroni correction are plotted in dark gray, and lags where the difference passed a $p<0.05$ uncorrected threshold are shown in light gray. Both analyses initially showed significant Bonferroni-corrected differences in Figure 7 of the main text. HR-BOLD cross-correlation significance tests were corrected for average heart rate, LF HRV, HF HRV, and RMSSD. CO_2-BOLD cross-correlations were corrected for average CO_2 and breathing rate.

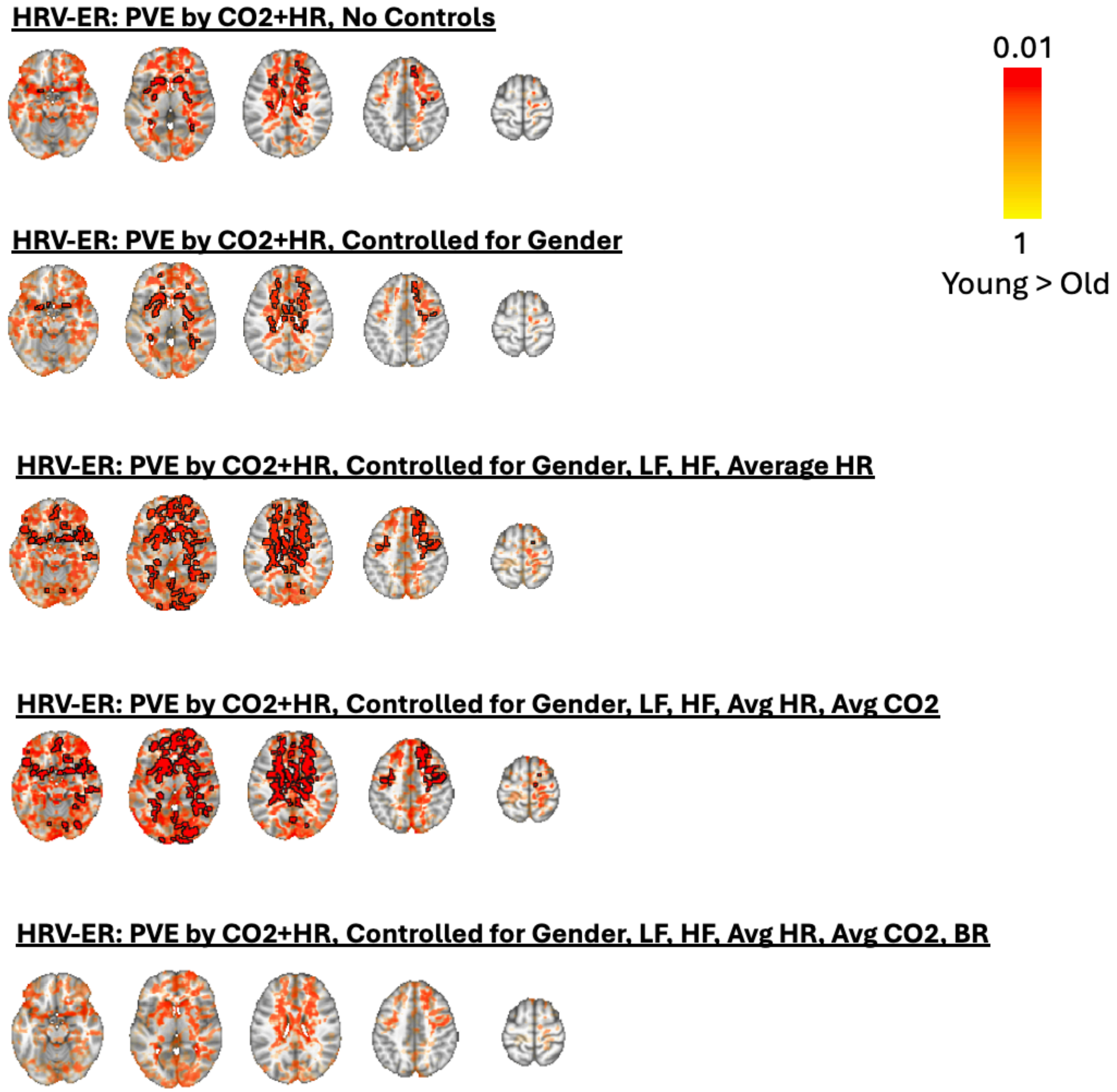

Supplemental Figure 7. Percent variance of BOLD signal explained by HR and CO_2 across all voxels in the HRV-ER dataset. Voxels in which percent variance explained in younger adults was statistically significantly greater than older adults ($p < 0.05$ TFCE-corrected) are outlined in black, and alpha-fading was used to highlight sub-threshold voxels.

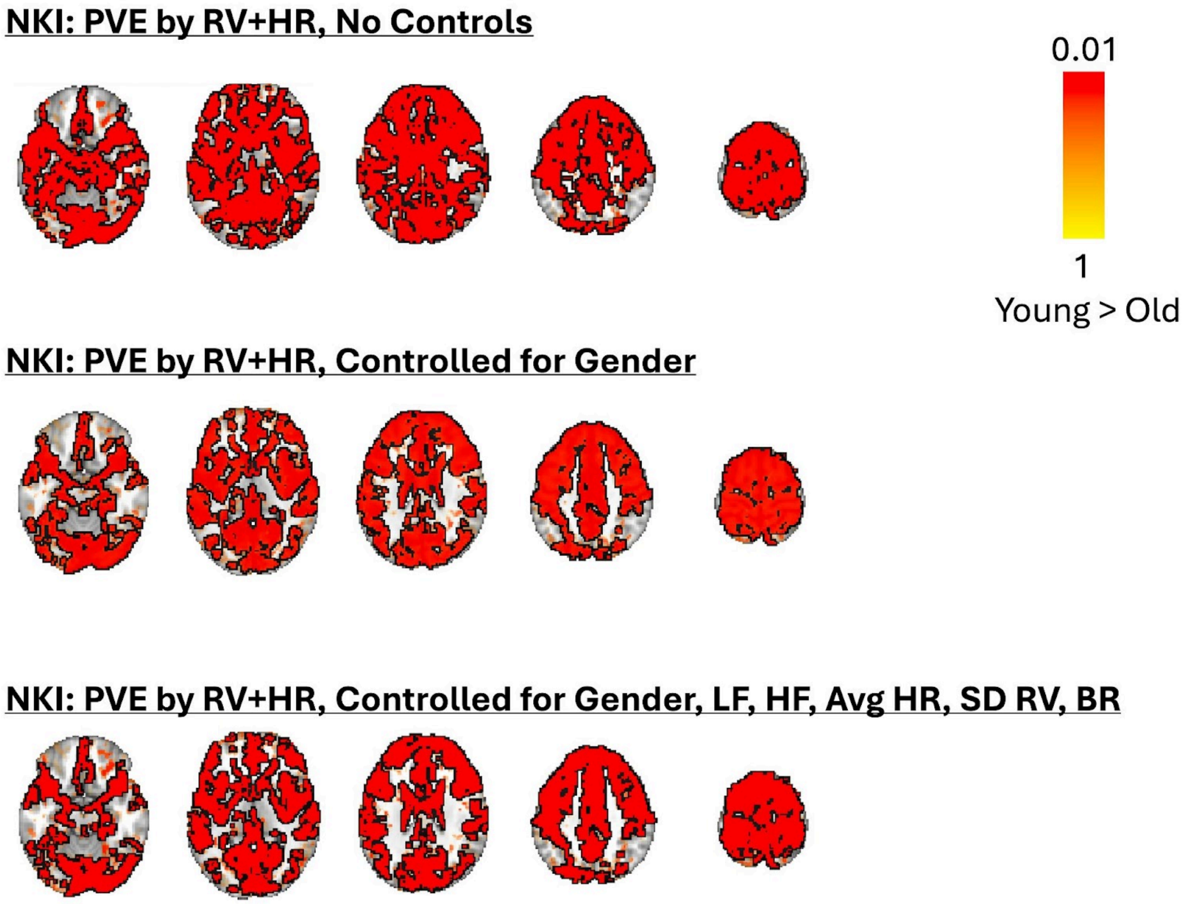

Supplemental Figure 8. Percent variance of BOLD signal explained by HR and RV across all voxels in the NKI dataset. Voxels in which percent variance explained in younger adults was statistically significantly greater than older adults (p < 0.05 TFCE-corrected) are outlined in black, and alpha-fading was used to highlight sub-threshold voxels.

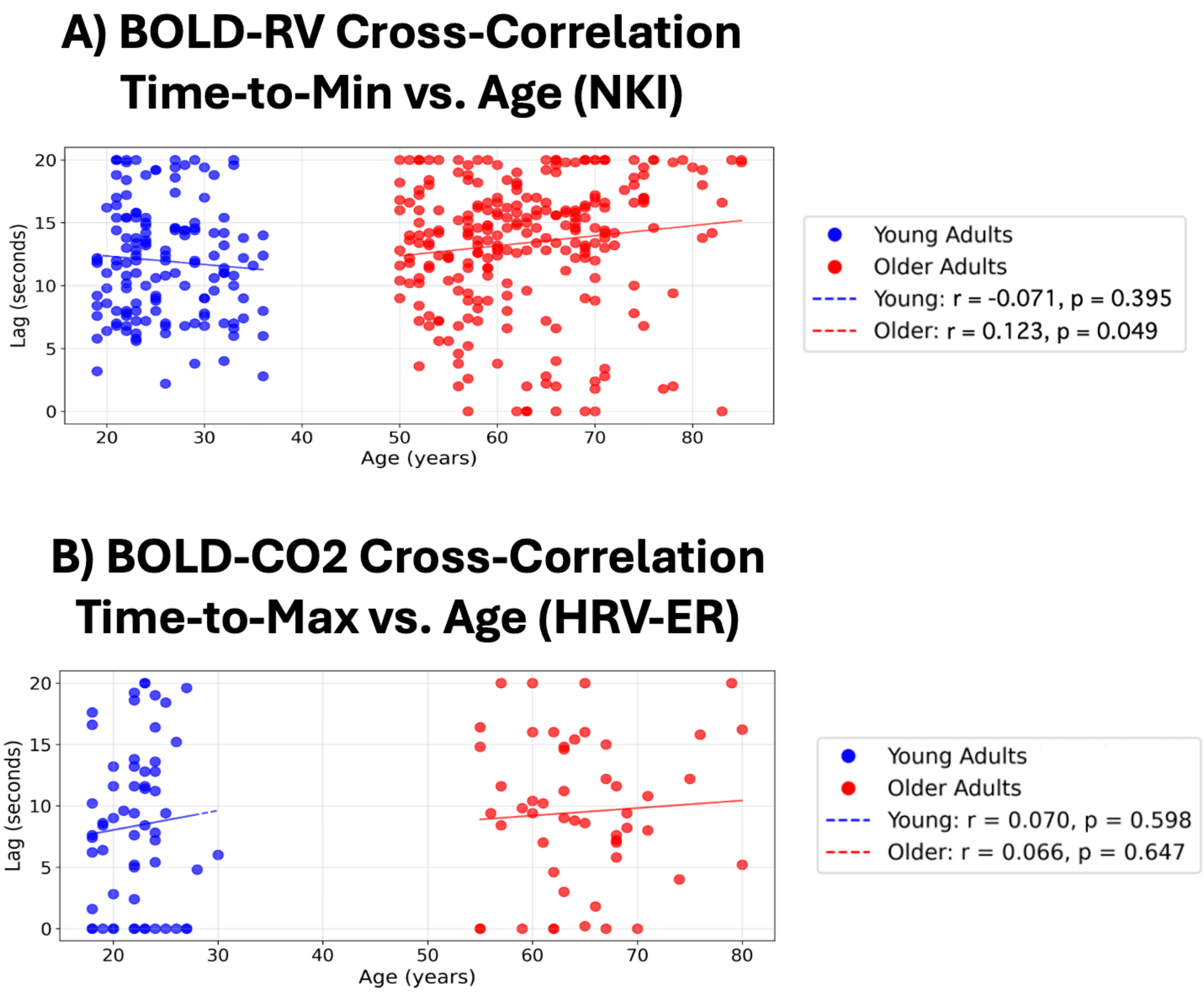

Supplemental Figure 9. A) BOLD-RV cross-correlation time-to-minimum (min) plotted against age in the NKI dataset B) BOLD-CO_2 cross-correlation time-to-maximum (max) plotted against age in the HRV-ER dataset. Pearson *r* and *p* values were determined for younger and older adults separately. The time-to-min/max lags were constrained to within 0 to 20 seconds for both datasets.
